# Cost-effectiveness of seasonal influenza vaccination of children in China: a modeling analysis

**DOI:** 10.1186/s40249-023-01144-6

**Published:** 2023-10-11

**Authors:** Qiang Wang, Huajie Jin, Liuqing Yang, Hui Jin, Leesa Lin

**Affiliations:** 1 Department of Epidemiology and Health Statistics, School of Public Health, Southeast University, Nanjing, 210009 China; 2 Department of Infectious Disease Epidemiology, London School of Hygiene and Tropical Medicine, London, WC1E 7TH UK; 3 King’s Health Economics, Institute of Psychiatry, Psychology and Neuroscience at King’s College London, London, SE5 8AF UK; 4 Centre for Digital Public Health in Emergencies, Institute for Risk and Disaster Reduction, University College London, London, WC1E 6BT UK; 5 Key Laboratory of Environmental Medicine Engineering, Ministry of Education, School of Public Health, Southeast University, Nanjing, 210009 China; 6 Laboratory of Data Discovery for Health (D24H), Hong Kong Science Park, Hong Kong, Hong Kong Special Administrative Region China; 7 WHO Collaborating Centre for Infectious Disease Epidemiology and Control, School of Public Health, LKS Faculty of Medicine, The University of Hong Kong, Hong Kong, Hong Kong Special Administrative Region China

**Keywords:** Influenza, Children, Vaccination, China, Cost-effectiveness analysis

## Abstract

**Background:**

China has a high burden of influenza-associated illness among children. We aimed to evaluate the cost-effectiveness of introducing government-funded influenza vaccination to children in China (fully-funded policy) compared with the status quo (self-paid policy).

**Methods:**

A decision tree model was developed to calculate the economic and health outcomes, from a societal perspective, using national- and provincial-level data. The incremental cost-effectiveness ratio (ICER) [incremental costs per quality-adjusted life year (QALY) gained] was used to compare the fully-funded policy with the self-paid policy under the willingness-to-pay threshold equivalent to national and provincial GDP per capita. Sensitivity analyses were performed and various scenarios were explored based on real-world conditions, including incorporating indirect effect into the analysis.

**Results:**

Compared to the self-paid policy, implementation of a fully-funded policy could prevent 1,444,768 [95% uncertainty range (UR): 1,203,446–1,719,761] symptomatic cases, 92,110 (95% UR: 66,953–122,226) influenza-related hospitalizations, and 6494 (95% UR: 4590–8962) influenza-related death per season. The fully-funded policy was cost-effective nationally (7964 USD per QALY gained) and provincially for 13 of 31 provincial-level administrative divisions (PLADs). The probability of a funded vaccination policy being cost-effective was 56.5% nationally, and the probability in 9 of 31 PLADs was above 75%. The result was most sensitive to the symptomatic influenza rate among children under 5 years [ICER ranging from − 25,612 (cost-saving) to 14,532 USD per QALY gained]. The ICER of the fully-funded policy was substantially lower (becoming cost-saving) if the indirect effects of vaccination were considered.

**Conclusions:**

Introducing a government-funded influenza policy for children is cost-effective in China nationally and in many PLADs. PLADs with high symptomatic influenza rate and influenza-associated mortality would benefit the most from a government-funded influenza vaccination program.

**Supplementary Information:**

The online version contains supplementary material available at 10.1186/s40249-023-01144-6.

## Background

Influenza-related disease burden among children is high globally. According to a recent systematic review, in 2018 there were 63.1–190.6 million influenza virus infections and 543,000–1,415,000 influenza-virus-associated hospital admissions due to acute lower respiratory infection among children aged under 5 years globally [1]. Vaccination is one of the most effective interventions to prevent influenza [2, 3]. Approximately one-third of 194 World Health Organization (WHO) Member States recommend influenza vaccination for children [4]. Seasonal influenza vaccination of children has been incorporated into the National Immunization Program (NIP) of many developed countries (e.g., the United Kingdom, Australia, and Republic of Korea) and some upper middle-income countries (e.g., Mexico) and lower middle-income countries (e.g., Bhutan) [5–9].

In China, there were more than 250 million children aged under 15 years in 2021, accounting for 12.5% of all children in that age group globally [10]. One study estimated that the average number of influenza cases among children aged 0–14 years in China was 3.6 million annually between 2010 and 2020 [11]. Childhood vaccination programs in China are divided into the Expanded Program on Immunization (EPI) and the non-EPI [12]. EPI vaccines are free and mandatory, while non-EPI vaccines, including influenza vaccine, also known are optional and billed. Children are prioritized to receive influenza vaccination [13]; however, under the current self-paid policy, influenza vaccination coverage among children in China is low, remaining at approximately 25% [14, 15]. Influenza vaccination uptake was higher in cities with stronger health resources, and that vaccination coverage was lower in less-developed provinces [16].

Additionally, as a non-EPI vaccine, the influenza vaccine is provided by the private sector [17, 18]. After a series of massive illegal private sector vaccine sales, which sharply decreased public trust in vaccines [19, 20], a new vaccine administration law was introduced in China in 2019, which recommended the gradual incorporation of vaccines sold on the private market into the NIP [21]. In the interim, provincial health authorities could introduce new vaccines to the immunization programs in their respective regions before they were included in the NIP at the national level [21]. Currently, at the local level, some cities (e.g., Beijing and Karamay) provide free influenza vaccination to children [17]. Evidence from Beijing has shown that a government-funded vaccination policy can improve influenza vaccine uptake (from 2 to 40%) [22, 23].

A government-funded vaccination policy could not only ease influenza burden by improving vaccine uptake, but could also increase public trust in vaccines. Importantly, a cost-effectiveness evaluation is needed to inform decision-makers of whether to publicly fund influenza vaccination at the national and provincial levels. Policy decisions at various levels are driven by uncertainties regarding the burden of influenza, as well as an unclear picture of the populations’ demographic structure and socioeconomic level [16]. Hence, the aim of this study was to evaluate the impact and cost-effectiveness of introducing influenza vaccination to children (fully-funded policy) compared with the status quo (self-paid policy) at the national and provincial levels in China.

## Methods

We compared the cost-effectiveness of government-funded influenza vaccination (fully-funded policy) with the status quo (self-paid policy) for children aged 6 months to 14 years in Chinese mainland from the societal perspective. In our study, we made the assumption that government-funded influenza vaccination would be free and optional for the pediatric population. This study was reported according to the updated Consolidated Health Economic Evaluation Reporting Standards Statement recommendations for reporting health economic evaluations.

### Decision tree model

**Fig. 1:**
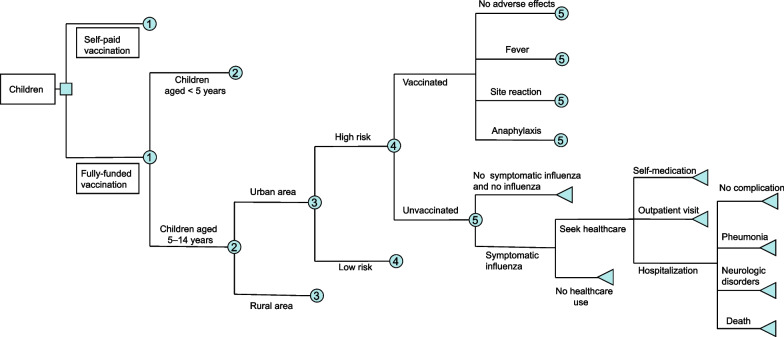
Simplified Decision Tree. Chance nodes labelled with the same number have the same subtree

A static decision-tree model (Fig. 1) was developed to estimate the cost and health outcomes that would result from using the alternative policy. The model was built using TreeAge Pro 2019 (TreeAge Software, Inc., Williamstown, MA, USA). In the model, children who are vaccinated are assumed to be at lower risk of developing influenza than unvaccinated children. Children who are vaccinated against influenza are at risk of developing side effects, including fever, site reactions, and anaphylaxis [24]. Of those children who are infected with influenza virus and become symptomatic, a proportion seek healthcare, including self-medication, outpatient visits, and hospitalization [16, 25]. Potential complications include pneumonia, neurological disorders, and death following hospitalization.

The time horizon of one influenza season (one year, starting in the 14th week of each year and ending in the 13th week of the following year) [26] was used in the model because most costs and outcomes caused by influenza, other than death due to influenza or its complications, occur within one influenza season. All life-years lost due to influenza-related deaths were included in the analysis. The unit costs were valued in 2019 Chinese Yuan (CNY). Where appropriate, unit costs were adjusted to 2019 values using the Consumer Price Index in China, then converted into 2019 US dollars (USD) (1 USD = 6.908 CNY) [27, 28]. Costs and quality-adjusted life-years (QALYs) were not discounted due to the short time horizon.

### Data sources

#### Study population

The US Centers for Disease Control and Prevention Advisory Committee on Immunization Practices has identified children aged < 5 years as being particularly vulnerable to influenza complications [24]. In China, different influenza vaccines are given to children aged < 3 years and ≥ 3 years [13]. The vaccine given to children aged < 3 years is named the “children’s type” and the vaccine given to children aged ≥ 3 years is named the “adults’ type.” We thus divided children under 5 years: 6 months to 2 years and 3–4 years to avoid the impact of differences of vaccines’ prices. These two age groups shared the same cost and epidemiological data except cost of vaccine. The population size of each age group was obtained from the National Bureau of Statistics in China [29]. Additionally, children were stratified according to urban and rural area of residence, in consideration of urban–rural differences in economic status and healthcare-seeking behaviors [16]. The proportion of children living in urban areas was obtained from the 2020 Population Census of China [30]. Population data are provided in the Additional file 1: Tables S1, S2.

According to the WHO influenza vaccination guidelines, children with underlying medical conditions, such as chronic respiratory disease and chronic cardiac disease, are at increased risk of hospitalization and death from influenza [31]. Hence, children were grouped into high-risk and low-risk groups in our model. The risk proportion was estimated using the method suggested by Clark et al., using data on the prevalence of each disease from the Global Burden of Diseases, Injuries, and Risk Factors Study 2017, to estimate the proportion of children with at least one underlying condition [32]. The detailed methods are reported in the Additional file 1: eMethods 1 and Tables S3).

#### Epidemiological data

Recently, Wang et al. estimated that, between 2010 and 2020, the mean incidence of influenza infection and symptomatic illness among children aged 0–14 years was 15.86 and 10.50 per 1000 person-seasons, respectively [11]. We applied this method to estimate rates of symptomatic influenza by age group and province in the model. The symptomatic rates for children aged < 5 years and aged ≥ 5 years were estimated to be 0.0586 and 0.0155, respectively, in the 2019–2020 season in China. Further details are provided in the Additional file 1: eMethods 2 and Table S4.

The healthcare-seeking rate among symptomatic cases was derived from a survey conducted in eastern China between 2011 and 2014 [25]. We used a healthcare-seeking rate of 84.4% in municipal districts as a proxy of that rate in urban areas, and a rate of 79.1% in counties as a proxy in rural areas. The proportions of different healthcare-seeking behaviors were obtained from a national survey conducted in 2017–2018 [33]. The proportions of complications after hospitalization were derived from two epidemiological surveys [34, 35]. Li et al. reported the influenza-associated excess respiratory mortality by provinces in China [36]. The case fatality ratio of hospitalization was calculated using influenza-associated mortality and other parameters. Detailed description was provided in the Additional file 1: eMethods 3 and Table S5.

According to one systematic review, children with influenza and underlying conditions have an increased risk of hospitalization [odds ratio (*OR*) = 3.39] and death (*OR*: = 2.04) compared to children with influenza without underlying conditions [37].

#### Vaccine related parameters

The cost of vaccination included the cost of vaccines, cost of administration, and cost of parent’s time taking children to be vaccinated [24, 38]. We searched the price of influenza vaccines on national and provincial official procurement websites from 2018 to 2021 (Additional file 1: Table S6). The prices of the child and adult influenza vaccine were estimated to be 4.56 USD and 6.66 USD, respectively, using bootstrap sampling of the data [16]. The cost of administration was derived from a national survey [39]. Additionally, the economic loss for parents due to taking their children to be vaccinated included productivity loss and transportation costs [39]. Productivity loss was estimated through multiplying the mean hourly wage of Chinese workers by average hour (1.43 h) spent [24, 39]. Detailed data regarding this calculation are provided in the supplementary material (Additional file 1: Table S7). We used the clinical effectiveness of influenza vaccine estimates of Tricco et al. in the model [40]. In China, most child and adult vaccines are inactivated split vaccines [13]. The odds ratios of the matched and mismatched trivalent inactivated influenza vaccine (TIV) effectiveness among the population were estimated to be 0.35 and 0.44, respectively. To obtain a conservative estimate of the effectiveness of vaccination, we used mismatched TIV effectiveness data among the general population as a proxy of effectiveness data for children.

Side-effect data were derived from the Chinese national surveillance system of adverse events of seasonal influenza vaccine during the 2015–2018 influenza season [41]. The cost of fever was assumed to be equal to the cost of self-medication. It was assumed that fever and site reaction do not cause health utility loss because of their short duration. The cost and utility loss due to anaphylaxis was obtained from published literature [24].

According to Yang et al. [17], we estimated influenza vacciantion coverage among children using number of vaccine doses supply (Additional file 1: eMethods 4 and Table S8). The influenza vaccination coverage among children in 2019–2020 season was estimated to 0.0695. Extrapolating from the experience in Beijing, vaccination coverage under the fully-funded policy was assumed to be 0.40.

#### Influenza-related disease burden

The cost of self-medication for pediatric influenza was derived from a survey in Jiangsu Province, China [42]. We assumed the cost of self-medication is the same for children aged < 5 years and ≥ 5 years. The cost of outpatient and inpatient services were based on a national survey, and included direct and indirect medical costs [43]. The cost of self-medication, outpatient services, and inpatient services for other provinces and at the national level were adjusted using the local ratio of gross domestic product (GDP) per capita in 2019.

The national- and provincial-level costs of influenza-related complications were obtained from a previous study [38]. The cost per inpatient case of Hib pneumonia was used as a proxy of the cost of an inpatient case of influenza pneumonia, and the cost per inpatient case of meningitis was used as a proxy of the cost of per inpatient case of neurologic disorder. The cost of death was assumed to be ten times the cost of one inpatient stay, according to a previous study [24]. The lifetime productivity loss caused by death was estimated using the human capita method [38]. The retirement age was set at 60 years. An annual discount rate of 3% was used in the estimation of lifetime productivity (Additional file 1: Table S9) [44].

The healthy utility loss for outpatients and inpatients was calculated using the method suggested by Yang et al. [16, 45]:

$${\text{QALY loss }} = \, \left( {{\text{utility of the general population }}{-}{\text{ utility of patients with influenza}}} \right) \, \times \, \left( {{\text{duration of illness}}/{365}.{25}} \right).$$

The utility of the general population was obtained from the National Health Services Surveys between 2008 and 2013 [46]. The utility of outpatients and inpatients with influenza aged < 5 years were estimated to be 0.6286 and 0.5900, respectively; those aged ≥ 5 years were estimated to be 0.6216 and 0.6132, respectively [45]. The mean duration of an influenza episode for outpatients and inpatients was assumed to be 6.2 days and 11.8 days, respectively [45]. The QALY loss for self-medicated patients was assumed to be 0.005. The QALY losses for pneumonia and neurologic disorders were derived from published literature [24].

#### Outcome measures

The primary outcomes of this study were total cost, effectiveness (QALY gained), and cost-effectiveness (incremental cost per QALY gained). The incremental cost-effectiveness ratio (ICER), defined as the incremental costs per QALY gained, was used to compare the fully-funded policy with the self-paid policy at the national and provincial levels (Additional file 1: eMethods 5). In the base-case analysis, the willingness-to-pay (WTP) threshold was set to USD 10,144, the GDP per capita in 2019 [47]. The GDP of each province is provided in the Additional file 1: Table S1. Additionally, we tested a WTP threshold estimated by University of York for China, which is between USD 1162 and USD 4595 per QALY [48].

#### Sensitivity analysis

To explore the drivers of the results, deterministic sensitivity analyses were performed of the base-case scenario at a national level. Probabilistic sensitivity analyses (PSA) were performed to examine the joint impact of parameter uncertainty on ICER using Monte Carlo simulation. The median values and the 95% uncertainty range (UR) (centiles 2.5–97.5) were estimated based on 10,000 simulations. The distributions of parameters are shown in Table 1. The cost-effectiveness acceptability curves were constructed under various WTP thresholds at a national and provincial level.

**Table 1:** Input parameters of the model

Parameters	Baseline value (range)	Distribution
Demographic parameters		
Number of age-specific children [29]	Additional file 1: Tables S1–2	NA
Proportion of age-specific children in the urban area [30]	Additional file 1: Tables S1–2	NA
Proportion of age-specific high-risk children [31, 32]	Additional file 1: Table S3	NA
Epidemiologic parameters		
Symptomatic influenza rate [11]	< 5 years: 0.0586 (0.0362–0.0976) 5–14 years: 0.0155 (0.0096–0.0256)	Beta
Proportion of cases seeking healthcare among symptomatic cases [25]	Urban: 0.844 (0.6752–1) Rural: 0.791 (0.6328–0.9492)	Beta
Proportion of outpatient among seeking healthcare cases [33]	< 5 years: 0.6976 (0.5581–0.8371) 5–14 years: 0.6579 (0.5263–0.7895)	NA
Proportion of hospitalization among seeking healthcare cases [33]	< 5 years: 0.0745 (0.0596–0.0894) 5–14 years: 0.0504 (0.0403–0.0605)	Beta
Proportion of complications and death		
Proportion of pneumonia [34, 35]	0.2017 (0.1614–0.2420)	Beta
Proportion of neurologic disorders [34, 35]	0.0017 (0.0014–0.0020)	Beta
Influenza-associated excess respiratory mortality [36]	Additional file 1: Table S5	Beta
Odds ratio of influenza-related hospitalization in high-risk groups compared to low-risk groups [37]	3.39 (2.60–4.42)	Lognormal
Odds ratio of influenza-related death in high-risk groups compared to low-risk groups [37]	2.04 (1.74–2.39)	Lognormal
Duration of influenza episode [45]	Outpatient: 6.2 (5.0–7.4) Hospitalization: 11.8 (9.4–14.2)	Normal
Vaccine related parameters		
Cost of vaccine	Children’s type: 4.56 (4.08–5.11) Adults’ type: 6.66 (4.70–9.74)	Gamma
Cost of administration [39]	3.52 (2.81–4.22)	Gamma
Cost of parent’s time to obtain the influenza vaccine in urban and rural areas [39]	Additional file 1: Table S7	NA
Odds ratio of vaccination effectiveness [40]	Matched: 0.35 (0.28–0.42) Mismatched: 0.44 (0.34–0.57)	Beta
Proportion of side effects		
Proportion of fever [41]	0.00004274	Beta
Proportion of injection site reaction [41]	0.00000854	Beta
Proportion of anaphylaxis [41]	0.00000753	Beta
Influenza vaccine coverage	Fully-funded policy: 0.400 Self-paid policy: 0.695	NA
Cost of illness and side effects		
Cost of self-medication [42]	High-risk children: 8.68 (6.944–10.416) Low-risk children: 6.06 (4.848, 7.272)	Gamma
Cost of outpatient [43]	< 5 years: 197.96 (158.38–237.56) 5–14 years: 154.53 (123.63–185.44)	Gamma
Cost of hospitalization [43]	< 5 years: 1523.12 (1218.55–1827.83) 5–14 years: 1431.21 (1145.02–1717.53)	Gamma
Cost of pneumonia [38]	1435.99 (1148.79–1723.19)	Gamma
Cost of neurologic disorders [38]	5270.80 (4216.64–6324.96)	Gamma
Cost of death [12]	< 5 years: 15,230.12 (12,184.10–18,276.14) 5–14 years: 14,310.21 (11,448.17–17,172.25)	Gamma
Discounted lifetime productivity [38]	Additional file 1: Table S9	NA
Cost of fever [42]	High-risk children: 8.68 (6.944–10.416) Low-risk children: 6.06 (4.848–7.272)	Gamma
Cost of injection site reaction [24]	77.53 (0–863.66)	Gamma
Cost of anaphylaxis [24]	1990.25 (65.76–17,388.08)	Gamma
Utilities		
Background health utility [46]	0.996 (0.953–1)	Beta
Utility of outpatient [45]	< 5 years: 0.6286 (0.5029–0.7543) 5–14 years: 0.6216 (0.4720–0.7080)	Beta
Utility of hospitalization [45]	< 5 years: 0.5900 (0.4973–0.7459) 5–14 years: 0.6132 (0.4906–0.7358)	Beta
Self-medication (QALY loss)	0.005 (0.001–0.01)	NA
Pneumonia (QALY loss) [24]	0.08 (0.054–0.1)	NA
Neurologic disorders (QALY loss) [24]	0.08 (0.054–0.1)	NA
Anaphylaxis (QALY loss) [24]	0.020 (0.006–0.041)	NA

*QALY* quality-adjusted life-years, *NA* not applicable

Additionally, we explored various scenarios in sensitivity analyses. In Scenario 1, we estimated the indirect effect of providing children with influenza vaccination based on previous epidemiological and modelling studies [49–52]. We used a conservative assumption that, when compared to a 0.0695 coverage (self-paid policy), symptomatic cases among children who were unvaccinated would decrease by 4% due to the indirect effect of a 40% vaccination coverage (fully-funded policy). We also analyzed the outcomes using a range of possible indirect effect values in the sensitivity analysis (1% and 10%). In Scenario 2, we used a conservative (30%) estimate of vaccination coverage under the fully-funded policy. In Scenario 3, we tested a higher probability of adverse events of vaccination based on a study conducted in the United States [24]. In Scenario 4, we tested the impact of switching to a healthcare sector perspective, as opposed to the societal perspective used in the base-case analysis. The influenza-related cost only included direct medical costs. The cost of parents taking their children to be vaccinated and the lifetime productivity loss were not considered in this scenario. In Scenario 5, we estimated the clinical effectiveness of vaccination based on the assumption of matched, rather than mismatched vaccines. In Scenario 6, we tested the joint impact of the indirect effect, higher probability of side effects, and mismatched vaccine effectiveness. A detailed description of the different scenarios is shown in Additional file 1: Table S10.

## Results

### Health and economic outcomes

Under implementation of a fully-funded policy, an estimated 98.95 million children aged 6 months to 14 years would be vaccinated against influenza nationally. Compared to the self-paid policy, the implementation of a fully-funded policy could prevent 1,444,768 (95% UR: 1,203,446–1,719,761) symptomatic cases, 92,110 (95% UR: 66,953–122,226) influenza-related hospitalizations, and 6494 (95% UR: 4590–8962) influenza-related deaths nationally in one influenza season (Table 2). We compared the incidence of hospitalization and death captured with the previous epidemiological data (Additional file 1: Table S11). Nationally, of the six scenarios, Scenario 1 (indirect effect) and Scenario 5 (matched vaccine) could avert a considerable number of symptomatic cases, hospitalizations, and deaths due to influenza compared to the base-case assumption (Table 2; Additional file 1: Table S12).

**Table 2:** Results of base case analysis and scenario analysis (fully-funded policy vs self-paid policy) (95% uncertainty range)

Scenario	Strategy	Total influenza symptomatic cases averted	Total influenza hospitalizati-ons averted	Total influenza death averted	Total cost (USD, million)	Incremental cost (USD, million)	Total QALYs	Incremental QALYs	ICER
Base-case	Fully-funded policy	1,444,768 (1,203,446–1,719,761)	92,110 (66,953–122,226)	6494 (4590–8962)	5927 (4600–7725)	123 (−350 to 485)	247,326,507 (247,285,841–247,353,414)	15,051 (8499–25,205)	7964 (cost-saving–45,356)
	Self-paid policy				5803 (4189–7995)		247,311,386 (247,260,803–247,344,681)		
Scenario 1	Fully-funded policy	1,633,628 (1,374,744–1,923,970)	104,748 (76,908–137,896)	7307 (5259–10,056)	5789 (4483–7512)	−13 (−518 to 386)	247,328,548 (247,288,031–247,355,018)	17,022 (9579–28,554)	Cost-saving (cost-saving–30,473)
	Self-paid policy				5792 (4183–7959)		247,311,651 (247,259,509–247,345,316)		
Scenario 2	Fully-funded policy	1,005,258 (839,277–1,196,052)	64,375 (46,762–86,090)	4525 (3197–6215)	5882 (4484–7730)	87 (−238 to 341)	247,322,686 (247,278,705–247,351,118)	10,388 (5981–17,493)	8183 (cost-saving–45,102)
	Self-paid policy				5794 (4200–7913)		247,312,330 (247,261,308–247,344,974)		
Scenario 3	Fully-funded policy	1,445,261 (1,201,100–1,712,829)	92,237 (67,297–123,245)	6476 (4629–8949)	5929 (4591–7726)	139 (−337 to 505)	247,326,784 (247,284,137–247,354,170)	15,057 (8546–25,231)	8787 (cost-saving–46,655)
	Self-paid policy				5795 (4157–8007)		247,311,680 (247,258,917–247,345,391)		
Scenario 4	Fully-funded policy	1,444,726 (1,201,191–1,719,733)	92,117 (66,950–122,304)	6486 (4592–8962)	1933 (1460–2866)	678 (422 to 889)	247,326,856 (247,287,021–247,353,147)	14,971 (8685–24,942)	44,673 (21,793–85,588)
	Self-paid policy				1240 (717–2385)		247,311,840 (247,262,261–247,344,057)		
Scenario 5	Fully- funded policy	1,672,660 (1,419,236–1,957,137)	107,649 (78,855–142,480)	7535 (5376–10,323)	5719 (4427–7434)	−48 (−571 to 358)	247,329,654 (247,289,887–247,355,467)	17,463 (9895–29,038)	Cost-saving (cost-saving–26,931)
	Self-paid policy				5763 (4133–7960)		247,312,198 (247,260,819–247,345,467)		
Scenario 6	Fully- funded policy	1,633,592 (1,370,504–1,930,172)	104,184 (76,035–138,828)	7367 (5234–10,110)	5805 (4522–7558)	0 (−531 to 403)	247,328,974 (247,288,475–247,354,283)	16,905 (9787–28,618)	Cost-saving (cost-saving–32,730)
	Self-paid policy				5801 (4170–8030)		247,312,094 (247,260,052–247,344,395)		

*QALY* quality-adjusted life-years, *USD* United States Dollar, *ICER* incremental costs per quality-adjusted life year (QALY) gained

**Fig. 2:**
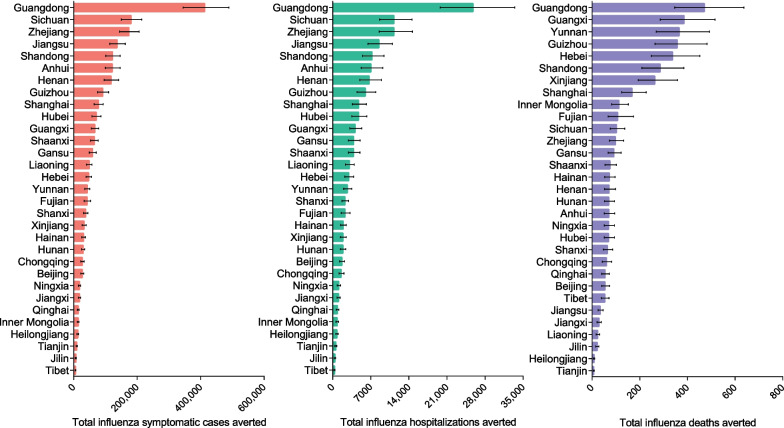
Health outcomes of fully-funded policy vs self-paid policy in different PLADs. PLADs: Provincial-level administrative divisions

Of the 31 provincial-level administrative divisions (PLADs) in Chinese mainland, Guangdong Province was associated with the largest number of symptomatic cases averted (412,410, 95% UR: 344,986–488,711), hospitalizations averted (25,797, 95% UR: 19,752–33,482), and deaths averted (471, 95% UR: 347–637) (Fig. 2).

### Cost-effectiveness analysis outcomes

Compared with the self-paid policy, the fully-funded policy could save 15,051 (95% UR: 8499–25,205) QALYs with an additional cost of USD 123 million (95%UR: − 350–485 million) (Table 2). The median ICER was estimated to be USD 7964 per QALY gained. Under the fully-funded policy, the economic cost and QALYs loss associated with death attributing to influenza accounted for the largest proportion of total economic cost (57%) and QALYs loss (44%) (Additional file 1: Fig. S1). The median ICER was lower than the GDP per capita in Scenario 1, 2 (low coverage under fully-funded policy), 3 (high proportion of side effects), 5, and 6 (mixed assumptions). The median ICER of the funded policy compared to the status quo was estimated to be USD 44,673 per QALY gained using a healthcare perspective (Scenario 4).

Among the 31 PLADs, the total cost of the fully-funded vaccination policy ranged from USD 22 million (Tibet) to USD 894 million (Guangdong Province) (Additional file 1: Table S13). The incremental QALYs gained from the fully-funded policy compared to the status quo ranged from 61 (Jilin Province) to 2693 (Guangdong Province). The fully-funded policy was associated with a cost-saving in 10 of 31 PLADs (Table 3). The ICER of the fully-funded policy was higher than the provincial GDP per capita in 18 PLADs, indicating that a fully-funded vaccination policy strategy would not be cost-effective in these PLADs.

**Table 3:** Cost-effectiveness analysis of influenza vaccination among children in various PLADs. PLADs: Provincial-level administrative divisions

Cost-effective analysis outcomes	PLADs
Cost-saving	Beijing; Guangdong; Hainan; Ningxia; Qinghai; Shanghai; Tianjin; Tibet; Xinjiang; Zhejiang
Cost-effective (< PLAD GDP per capita threshold)	Inner Mongolia; Jiangsu; Guizhou
Not cost-effective	Anhui; Chongqing; Fujian; Gansu; Guangxi; Hebei; Heilongjiang; Henan; Hubei; Hunan; Jiangxi; Jilin; Liaoning; Shaanxi; Shandong; Shanxi; Sichuan; Yunnan

### The impact of variables changing on cost-effectiveness analysis

The results of the one-way sensitivity analyses are summarized in Additional file 1: Fig. S2. The results were sensitive to the assumptions regarding the symptomatic influenza rate among children, vaccination effectiveness, influenza-associated excess respiratory mortality, vaccine price, proportion of high-risk children, proportion of children with influenza seeking healthcare, and cost of administration. Implementing a fully-funded policy would not be considered cost-effective if the symptomatic influenza rate among children aged < 5 years falls below 0.0520.

**Fig. 3:**
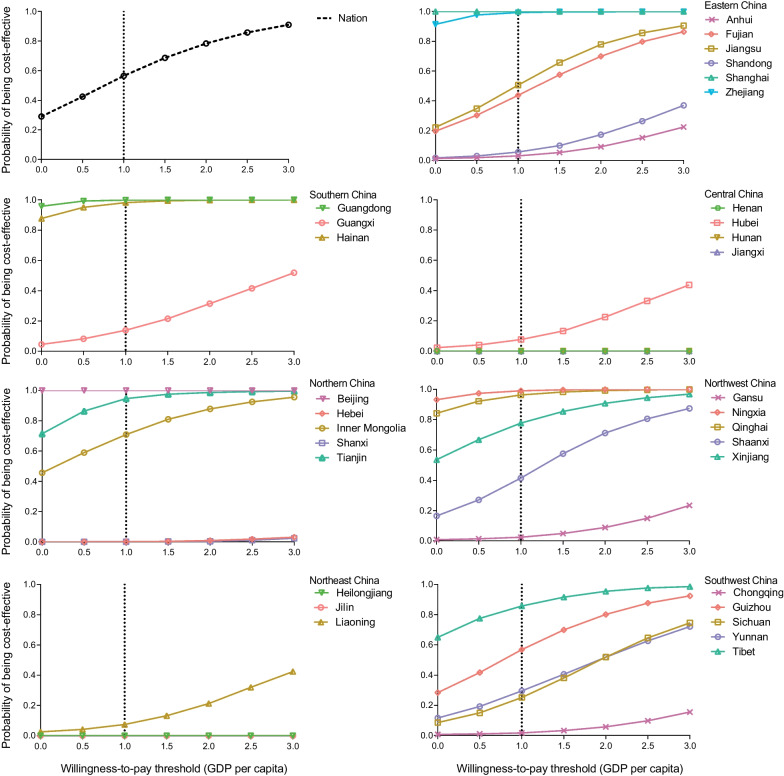
Cost-effectiveness acceptability curves at national and provincial level. One unit is 0.5 times GDP per capita

For most of PLADs, the cost-effective results were highly sensitive to the range of province-specific influenza-associated mortality and symptomatic influenza rate among children (Additional file 1: Table S14). At a provincial level, the probability of a fully-funded policy being cost-effective was > 75% in 9 of 31 PLADs, and < 50% in 20 PLADs, based on a WTP threshold of the provincial GDP per capita (Fig. 3). Moreover, the probability of a fully-funded vaccination policy being cost-effective was < 1% in Jilin, Heilongjiang, Jiangxi, Henan, Hunan, Hebei, and Shanxi Provinces. The detailed PSA results at a provincial level using the WTP threshold of Woods et al. are shown in Additional file 1: Fig. S3.

#### Cost-effective probability of the fully-funded policy

The PSA showed that the probability of a fully-funded vaccination policy being cost-effective was 56.5% at a WTP threshold of GDP per capita, and 41.1% at a WTP threshold of USD 4595 suggested by Woods et al. (Additional file 1: Figs. S3, S4). The PSA indicated that, with the threshold of GDP per capita, the probability of the fully-funded vaccination policy being cost-effective in Scenarios 1, 5, and 6 was more than 60% (Additional file 1: Figs. S4, S5). The probability of the fully-funded policy being cost-effective was similar to that of the base case, in Scenarios 2 and 3 at a WTP threshold of the GDP per capita.

## Discussion

This study comprehensively evaluated the cost-effectiveness of introducing a fully-funded influenza vaccination program to Chinese children at the national and provincial levels. The results show that, at a WTP threshold of the GDP per capita, a policy of influenza vaccination funded by the government would be cost-effective nationally and for 42% (13/31) of PLADs.

Three previous studies have assessed the cost-effectiveness of influenza vaccination for children in China, and all of them found influenza vaccination to be cost-effective [53–55]. However, none of the previous studies assessed the cost-effectiveness of fully-funded influenza vaccine for children under 15 years old in all PLADs in China. Zhou et al. reported that, from a healthcare perspective, compared with no vaccination, the cost-effective ratio of influenza vaccination for children aged 6–59 months and 60 months to 14 years would be USD 0 and 37 per case averted, respectively, in four provinces [53]. Another study demonstrated that, at the WTP of three-fold GDP per capita, quadrivalent influenza vaccine would be more cost-effective than TIV for school children in Beijing City [54]. A study by Xiang et al. showed that providing two doses of vaccine to preschool children in child nursery settings would cost 178.77 CNY (25.88 USD) per influenza case averted [55]. However, some limitations, including simple model assumptions, did exist in these studies. To our knowledge, this study is the first comprehensive evaluation of the cost-effectiveness of influenza vaccination of children in China.

Our findings show that, at the national level, providing free influenza vaccination to children aged 6 months to14 years would be cost-effective compared to the current self-paid policy. At the provincial level, fully-funded influenza vaccination would be cost-effective in 13 PLADs. Moreover, less socioeconomically developed PLADs, including Tibet, Qinghai, Xinjiang, and Ningxia, could benefit from the implementation of the fully-funded policy. We suggest that, given the limited opportunities to access vaccines in the private sector and the high influenza-associated burden of these less-developed regions, providing an influenza vaccination to children that is fully-funded by the government would provide a valuable opportunity to promote health equity.

Cost-effectiveness outcomes varied by PLAD. The probability of a fully-funded vaccination policy being cost-effective was > 50% in nearly one-third of PLADs, which might result from the high influenza burden. The intensive influenza virus activities (high symptomatic rate) or insufficient medical resources (high influenza-associated mortality) might explain the high influenza burden in these PLADs. However, the probability of a fully-funded vaccination policy being cost-effective was < 1% in Jilin, Heilongjiang, Jiangxi, Henan, Hunan, Hebei, and Shanxi Provinces. We believe that the relatively low symptomatic influenza rate estimation and low influenza-associated excess respiratory mortality in these provinces might be the main factors making them less likely to benefit from a fully-funded influenza vaccination policy. Currently, the implementation of government-funded influenza policy is not likely to be recommended in these PLADs, given the cost-effective results. However, the symptomatic influenza rate and low influenza-associated mortality might vary across influenza seasons; therefore, enhancing influenza surveillance, particularly in less-developed PLADs, is a priority that should be given greater attention to provide more available and reliable evidence. Additionally, low economic cost associated with outpatient and inpatient services in these PLADs might contribute to the low probability of being cost-effective.

Various scenarios were explored to test the robustness of these findings. Noticeably, the analysis from a healthcare sector perspective (Scenario 4) found that the probability of the fully-funded policy being cost-effective was low under WTP threshold. This is because the study population were pediatric population, which indicated the discounted lifetime productivity loss associated with death might account for a large proportion of economic cost.

This study has some limitations, and most are related to the limited evidence availability. For example, if there was a lack of data at the provincial level, we either applied national data across different PLADs (e.g., symptomatic rate and healthcare-seeking rate), or adjusted national data based on the local GDP (e.g., treatment cost). Second, all influenza-related deaths were assumed to occur in hospitals. It is likely that this underestimated the ICER because some influenza-related deaths occur outside health facilities. Third, due to low coverage, we assumed that none of the children in the target cohort have received vaccination previously, and therefore would require two doses of vaccines; in reality, some children might have already received one dose of vaccine when then entered into our model and therefore would only require one dose of vaccine [13]. Fourth, the retirement age used to calculate the discounted lifetime productivity loss was set at 60 years, which might be influenced by sex and type of jobs. Fifth, dynamic transmission modelling might be technically more appropriate in modelling the disease transmission process for infectious diseases. Recent cost-effectiveness evaluations of influenza vaccination have placed significant emphasis on the indirect effects that arise when a population is vaccinated [56, 57]. The parameter has been estimated and applied using a dynamic transmission model [56, 57]. Zhang et al. estimated the effectiveness indirectly using a regression model based on various rates of Hib vaccination [38]. However, a lack of data limited our ability to estimate the indirect effect of influenza vaccination in the present study. We conservatively used a fixed value as a proxy of the indirect effectiveness at 0.40 coverage compared with 0.0695 coverage. Additionally, we performed sensitivity analyses of the indirect effects, which indicated that small indirect effects could increase the cost-effectiveness of a fully-funded policy. Sixth, our analysis encountered limitations as we were unable to explore the healthcare payer or patient perspective, primarily due to the unavailability of data on reimbursement rates for pediatric flu vaccines at the national and provincial levels. Finally, our findings from this model were sensitive to the assumptions, especially symptomatic influenza rates, vaccination effectiveness, and influenza-associated excess respiratory mortality, which meant fully-funded policy being cost-effective should be interpreted cautiously.

## Conclusions

Our study, using national and province-specific influenza disease burden and demographic information, found that a fully funded influenza vaccination policy for Chinese children would be cost-effective, both nationally and in 13 of 31 PLADs. For less socioeconomically developed provinces with limited vaccine accessibility and a high burden of influenza-associated disease, implementing a fully funded influenza vaccination policy would provide a valuable opportunity to promote health equity.

### Supplementary Information

**Additional file 1**: **Table S1**. Number of age-specific children in China in 2019. **Table S2**. Proportion of urban population in China. eMethods 1. **Table S3**. Risk conditions definition and estimated high-risk proportions of age-specific children. eMethods 2. **Table S4** Number of positive specimens and estimated influenza symptomatic rate. eMethods 3. **Table S5**. Influenza-associated excess respiratory mortality at national-and-provincial level. **Table S6**. Prices of vaccines. **Table S7**. Estimated parents’ cost due to bringing child to vaccination clinics (USD). eMethods 4. **Table S8**. Estimated influenza vaccine coverage at national-and-provincial level. **Table S9**. Estimated lifetime productivity loss due to influenza related-death (USD). eMethods 5. **Table S10**. Different scenarios assumed. **Table S11**. Comparisons of influenza burden between our findings and other studies. **Table S12**. Comparison of costs and health outcome of different indirect effect values. **Figure S1**. Economic cost and QALY loss caused by different outcomes. **Table S13**. Comparison of costs and health outcome of vaccination strategies in various provinces. **Figure S2**. One-way sensitivity analyses for the most influential model parameters on ICER (USD/QALY gained). **Table S14**. One-way sensitivity analyses for the five most influential model parameters on ICER at the provincial level (USD/QALY gained). **Figure S3**. Cost-effectiveness acceptability curves at national and provincial level (Woods et al. threshold). **Figure S4**. Monte Carlo simulation results in various scenarios. **Figure S5**. Cost-effectiveness acceptability curves in various scenarios.

## Data Availability

All data generated or analysed during this study are included in this published article [and its Additional files].

## Abbreviations

95% UR: 95% Uncertainty range
CNY: Chinese Yuan Renminbi
EPI: Expanded Program on Immunization
GDP: Gross domestic product
Hib: Haemophilus influenzae type b
ICER: Incremental cost-effectiveness ratio
NIP: National Immunization Program
*OR*: Odds ratio
QALYs: Quality-adjusted life-years
PSA: Probabilistic sensitivity analyses
TIV: Inactivated influenza vaccine
USD: US dollars
WHO: World Health Organization
WTP: Willingness-to-pay
PLADs: Provincial-level administrative divisions

## References

[1] Wang X, Li Y, O'Brien KL, Madhi SA, Widdowson MA, Byass P (2020). Global burden of respiratory infections associated with seasonal influenza in children under 5 years in 2018: a systematic review and modelling study. Lancet Glob Health.

[2] Claeys C, Zaman K, Dbaibo G, Li P, Izu A, Kosalaraksa P (2018). Prevention of vaccine-matched and mismatched influenza in children aged 6–35 months: a multinational randomised trial across five influenza seasons. Lancet Child Adolesc Health.

[3] Rolfes MA, Flannery B, Chung JR, O'Halloran A, Garg S, Belongia EA (2019). Effects of influenza vaccination in the United States during the 2017–2018 influenza season. Clin Infect Dis.

[4] Ortiz JR, Perut M, Dumolard L, Wijesinghe PR, Jorgensen P, Ropero AM (2016). A global review of national influenza immunization policies: analysis of the 2014 WHO/UNICEF Joint Reporting Form on immunization. Vaccine.

[5] Department of Health, Australian Government. National Immunisation Program Schedule. 2023. https://www.health.gov.au/health-topics/immunisation/when-to-get-vaccinated/national-immunisation-program-schedule. Accessed 9 Sept 2023.

[6] European Centre for Disease Prevention and Control. Seasonal influenza vaccination and antiviral use in EU/EEA Member States: Overview of vaccination recommendations for 2017–2018 and vaccination coverage rates for 2015–2016 and 2016–2017 influenza seasons. 2018. https://www.ecdc.europa.eu/sites/default/files/documents/Seasonal-influenza-antiviral-use-EU-EEA-Member-States-December-2018_0.pdf. Accessed 2 Jan 2022.

[7] Yun JW, Noh JY, Song JY, Chun C, Kim Y, Cheong HJ (2017). The Korean influenza national immunization program: history and present status. Infect Chemother.

[8] Tapia-Conyer R, Betancourt-Cravioto M, Montoya A, Falcón-Lezama JA, Alfaro-Cortes MM, Saucedo-Martínez R (2021). A call for a reform of the influenza immunization program in Mexico: epidemiologic and economic evidence for decision making. Vaccines (Basel).

[9] Regional Office for South-East Asia, World Health Organization. Bhutan factsheet 2020: expanded programme on Immunization (EPI). World Health Organization. Regional Office for South-East Asia. 2020. https://apps.who.int/iris/handle/10665/336755. Accessed 2 Jun 2022.

[10] United Nations. World population Prospects. 2022. https://population.un.org/wpp/Download/Standard/CSV/. Accessed 9 Sept 2023.

[11] Wang Q, Yang L, Liu C, Jin H, Lin L (2022). Estimated incidence of seasonal influenza in China from 2010 to 2020 using a multiplier model. JAMA Netw Open.

[12] He Y, Liu Y, Dai B, Zhao L, Lin J, Yang J (2021). Assessing vaccination coverage, timeliness, and its temporal variations among children in a rural area in China. Hum Vaccin Immunother.

[13] National Immunization Advisory Committee (NIAC) Technical Working Group (TWG) Influenza Vaccination TWG. Zhonghua Liu Xing Bing Xue Za Zhi. 2020;41(10):1555–1576. 10.3760/cma.j.cn112338-20200904-01126 (in Chinese). 10.3760/cma.j.cn112338-20200904-01126 33297613

[14] Zhou L, Su Q, Xu Z, Feng A, Jin H, Wang S (2013). Seasonal influenza vaccination coverage rate of target groups in selected cities and provinces in China by season (2009/10 to 2011/12). PLoS ONE.

[15] Wang Q, Yue N, Zheng M, Wang D, Duan C, Yu X (2018). Influenza vaccination coverage of population and the factors influencing influenza vaccination in mainland China: a meta-analysis. Vaccine.

[16] Yang J, Atkins KE, Feng L, Baguelin M, Wu P, Yan H (2020). Cost-effectiveness of introducing national seasonal influenza vaccination for adults aged 60 years and above in mainland China: a modelling analysis. BMC Med.

[17] Yang J, Atkins KE, Feng L, Pang M, Zheng Y, Liu X (2016). Seasonal influenza vaccination in China: landscape of diverse regional reimbursement policy, and budget impact analysis. Vaccine.

[18] Zheng Y, Rodewald L, Yang J, Qin Y, Pang M, Feng L (2018). The landscape of vaccines in China: history, classification, supply, and price. BMC Infect Dis.

[19] Parry J (2016). Crackdown on illegal vaccine sales in China leads to 37 arrests. BMJ.

[20] Wang X, Lin L, Xu J, Wang W, Zhou X (2021). Expectant parents' vaccine decisions influenced by the 2018 Chinese vaccine crisis: a cross-sectional study. Prev Med.

[21] The State Council of the People's Republic of China. Vaccine administration law of the people's Republic of China. 2019. http://www.gov.cn/xinwen/2019-06/30/content_5404540.htm. Accessed 30 Jun 2021.

[22] Wu J, Dong ZY, Ding LX, Liu HL (2005). Influenza vaccination practice in Beijing during 1999–2004. J Pub Health Prev Med.

[23] Lv M, Fang R, Wu J, Pang X, Deng Y, Lei T (2016). The free vaccination policy of influenza in Beijing, China: the vaccine coverage and its associated factors. Vaccine.

[24] Hart RJ, Stevenson MD, Smith MJ, LaJoie AS, Cross K (2018). Cost-effectiveness of strategies for offering influenza vaccine in the Pediatric Emergency Department. JAMA Pediatr.

[25] Zhang T, Zhang J, Hua J, Wang D, Chen L, Ding Y (2016). Influenza-associated outpatient visits among children less than 5 years of age in eastern China, 2011–2014. BMC Infect Dis.

[26] Chinese National Influenza Center. National Influenza Surveillance Technical Guide (2017 Edition). 2017. http://ivdc.chinacdc.cn/cnic/zyzx/jcfa/201709/t20170930_153976.htm. Accessed 1 Dec 2021.

[27] Organization for Economic Co-operation and Development. Exchange rates. 2023. https://data.oecd.org/conversion/exchange-rates.htm#indicator-chart. Accessed 9 Sept 2023.

[28] National Bureau of Statistics of China. Consumer Price Index. 2023. http://data.stats.gov.cn/english/easyquery.htm?cn=C01. Accessed 9 Sept 2023.

[29] National Bureau of Statistics of China. Population size. 2023. https://data.stats.gov.cn/easyquery.htm?cn=C01. Accessed 9 Sept 2023.

[30] National Bureau of Statistics of China. China Population Census Yearbook 2020. http://www.stats.gov.cn/sj/pcsj/rkpc/7rp/indexch.htm. Accessed 9 Sept 2023.

[31] World Health Organization. Global epidemiological surveillance standards for influenza. 2013. https://www.who.int/publications/i/item/9789241506601. Accessed 19 May 2022.

[32] Clark A, Jit M, Warren-Gash C, Guthrie B, Wang HHX, Mercer SW (2020). Global, regional, and national estimates of the population at increased risk of severe COVID-19 due to underlying health conditions in 2020: a modelling study. Lancet Glob Health.

[33] Ren X, Geoffroy E, Tian K, Wang L, Feng L, Feng J (2019). Knowledge, attitudes, and behaviors (KAB) of influenza vaccination in china: a cross-sectional study in 2017/2018. Vaccines (Basel).

[34] Huai Y, Guan X, Liu S, Uyeki TM, Jiang H, Klena J (2017). Clinical characteristics and factors associated with severe acute respiratory infection and influenza among children in Jingzhou. China Influenza Other Respir Viruses.

[35] Shi Y, Chen W, Zeng M, Shen G, Sun C, Liu G (2021). Clinical features and risk factors for severe influenza in children: a study from multiple hospitals in Shanghai. Pediatr Neonatol.

[36] Li L, Liu Y, Wu P, Peng Z, Wang X, Chen T (2019). Influenza-associated excess respiratory mortality in China, 2010–15: a population-based study. Lancet Public Health.

[37] Mertz D, Kim TH, Johnstone J, Lam PP, Science M, Kuster SP (2013). Populations at risk for severe or complicated influenza illness: systematic review and meta-analysis. BMJ.

[38] Zhang H, Garcia C, Yu W, Knoll MD, Lai X, Xu T (2021). National and provincial impact and cost-effectiveness of Haemophilus influenzae type b conjugate vaccine in China: a modeling analysis. BMC Med.

[39] Yu W, Lu M, Wang H, Rodewald L, Ji S, Ma C (2018). Routine immunization services costs and financing in China, 2015. Vaccine.

[40] Tricco AC, Chit A, Soobiah C, Hallett D, Meier G, Chen MH (2013). Comparing influenza vaccine efficacy against mismatched and matched strains: a systematic review and meta-analysis. BMC Med.

[41] Wu WD, Li KL, Xu DS, Ye JK, Xiao QY, Wang HQ (2019). Study on surveillance data of adverse events following immunization of seasonal influenza vaccine in China during 2015–2018 influenza season. Zhonghua Yu Fang Yi Xue Za Zhi.

[42] Wang Y, Chen L, Cheng F, Biggerstaff M, Situ S, Zhou S (2021). Economic burden of influenza illness among children under 5 years in Suzhou, China: report from the cost surveys during 2011/12 to 2016/17 influenza seasons. Vaccine.

[43] Yang J, Jit M, Leung KS, Zheng YM, Feng LZ, Wang LP (2015). The economic burden of influenza-associated outpatient visits and hospitalizations in China: a retrospective survey. Infect Dis Poverty.

[44] Walker DG, Hutubessy R, Beutels P (2010). WHO Guide for standardisation of economic evaluations of immunization programmes. Vaccine.

[45] Yang J, Jit M, Zheng Y, Feng L, Liu X, Wu JT (2017). The impact of influenza on the health related quality of life in China: an EQ-5D survey. BMC Infect Dis.

[46] Yao Q, Liu C, Zhang Y, Xu L (2019). Changes in health-related quality of life of Chinese populations measured by the EQ-5D-3 L: a comparison of the 2008 and 2013 National Health Services Surveys. Health Qual Life Outcomes.

[47] National Bureau of Statistics of China. GDP Report. 2023. https://data.stats.gov.cn/easyquery.htm?cn=C01. Accessed 9 Sept 2023.

[48] Woods B, Revill P, Sculpher M, Claxton K (2016). Country-level cost-effectiveness thresholds: initial estimates and the need for further research. Value Health.

[49] King JC, Stoddard JJ, Gaglani MJ, Moore KA, Magder L, McClure E (2006). Effectiveness of school-based influenza vaccination. N Engl J Med.

[50] Loeb M, Russell ML, Moss L, Fonseca K, Fox J, Earn DJ (2010). Effect of influenza vaccination of children on infection rates in Hutterite communities: a randomized trial. JAMA.

[51] Yin JK, Heywood AE, Georgousakis M, King C, Chiu C, Isaacs D (2017). Systematic review and meta-analysis of indirect protection afforded by vaccinating children against seasonal influenza: implications for policy. Clin Infect Dis.

[52] Eichner M, Schwehm M, Eichner L, Gerlier L (2017). Direct and indirect effects of influenza vaccination. BMC Infect Dis.

[53] Zhou L, Situ S, Feng Z, Atkins CY, Fung IC, Xu Z (2014). Cost-effectiveness of alternative strategies for annual influenza vaccination among children aged 6 months to 14 years in four provinces in China. PLoS ONE.

[54] Zhu D, Lv M, Bai Y, Wu J, He P (2022). Cost-effectiveness analysis of quadrivalent seasonal influenza vaccines in Beijing: a modeling analysis. Vaccine.

[55] Xiang Y, Zhou D, Zhong J, Yu X, Chen W (2021). Economic burden of influenza like outbreaks in childcare settings and health economic evaluation of influenza vaccines in Yuexiu District, Guangzhou South China. J Prev Med.

[56] Sandmann FG, van Leeuwen E, Bernard-Stoecklin S, Casado I, Castilla J, Domegan L (2022). Health and economic impact of seasonal influenza mass vaccination strategies in European settings: a mathematical modelling and cost-effectiveness analysis. Vaccine.

[57] de Boer PT, Nagy L, Dolk FCK, Wilschut JC, Pitman R, Postma MJ (2021). Cost-effectiveness of pediatric influenza vaccination in The Netherlands. Value Health.

