# Supplementary material for: Cost-effectiveness of seasonal influenza vaccination of children in China: a modeling analysis
Source: Infect Dis Poverty. 2023 Oct 11;12:92. doi: 10.1186/s40249-023-01144-6 (PMC10566174; doi:10.1186/s40249-023-01144-6)
Supplement: Supplementary file 1 — Additional file 1: Table S1. Number of age-specific children in China in 2019. Table S2. Proportion of urban population in China. eMethods 1. Table S3. Risk conditions definition and estimated high-risk proportions of age-specific children. eMethods 2. Table S4 Number of positive specimens and estimated influenza symptomatic rate. eMethods 3. Table S5. Influenza-associated excess respiratory mortality at national-and-provincial level. Table S6. Prices of vaccines. Table S7. Estimated parents’ cost due to bringing child to vaccination clinics (USD). eMethods 4. Table S8. Estimated influenza vaccine coverage at national-and-provincial level. Table S9. Estimated lifetime productivity loss due to influenza related-death (USD). eMethods 5. Table S10. Different scenarios assumed. Table S11. Comparisons of influenza burden between our findings and other studies. Table S12. Comparison of costs and health outcome of different indirect effect values. Figure S1. Economic cost and QALY loss caused by different outcomes. Table S13. Comparison of costs and health outcome of vaccination strategies in various provinces. Figure S2. One-way sensitivity analyses for the most influential model parameters on ICER (USD/QALY gained). Table S14. One-way sensitivity analyses for the five most influential model parameters on ICER at the provincial level (USD/QALY gained). Figure S3. Cost-effectiveness acceptability curves at national and provincial level (Woods et al. threshold). Figure S4. Monte Carlo simulation results in various scenarios. Figure S5. Cost-effectiveness acceptability curves in various scenarios. [file 40249_2023_1144_MOESM1_ESM.docx]

**Cost-effectiveness of seasonal influenza vaccination of children in China: a modeling analysis**

Table S1. Number of age-specific children in China in 2019

Table S2. Proportion of urban population in China

eMethods 1

Table S3. Risk conditions definition and estimated high-risk proportions of age-specific children

eMethods 2

Table S4 Number of positive specimens and estimated influenza symptomatic rate

eMethods 3

Table S5. Influenza-associated excess respiratory mortality at national-and-provincial level

Table S6. Prices of vaccines

Table S7. Estimated parents’ cost due to bringing child to vaccination clinics (USD)

eMethods 4

Table S8. Estimated influenza vaccine coverage at national-and-provincial level

Table S9. Estimated lifetime productivity loss due to influenza related-death (USD)

eMethods 5

Table S10. Different scenarios assumed

Table S11. Comparisons of influenza burden between our findings and other studies

Table S12. Comparison of costs and health outcome of different indirect effect values

Figure S1. Economic cost and QALY loss caused by different outcomes

Table S13. Comparison of costs and health outcome of vaccination strategies in various provinces

Figure S2. One-way sensitivity analyses for the most influential model parameters on ICER (USD/QALY gained)

Table S14. One-way sensitivity analyses for the five most influential model parameters on ICER at the provincial level (USD/QALY gained)

Figure S3. Cost-effectiveness acceptability curves at national and provincial level (Woods et al. threshold)

Figure S4. Monte Carlo simulation results in various scenarios

Figure S5. Cost-effectiveness acceptability curves in various scenarios

**Table S1. Number of age-specific children in China in 2019**

|  | **Number of 6 m****onths–2 years children** | **Number of 3–4 years children** | **Number of 5–14 years children** | **Number of 6 month–14 years children** | **Proportion of 6 months–2 years children in 6 months–4 years children** | **Proportion of 6 months–4 years children in 6 months–14 years children** | **GDP per capita (USD)** |
| --- | --- | --- | --- | --- | --- | --- | --- |
| Nation | 35,644,598 | 36,245,262 | 175,500,050 | 247,389,910 | 49.58% | 29.06% | 10,144 |
| Beijing | 465,116 | 474,769 | 1,575,257 | 2,515,142 | 49.49% | 37.37% | 23,822 |
| Tianjin | 278,399 | 289,092 | 1,259,041 | 1,826,532 | 49.06% | 31.07% | 13,037 |
| Hebei | 1,915,477 | 1,973,423 | 10,896,351 | 14,785,251 | 49.25% | 26.30% | 6685 |
| Shanxi | 817,054 | 824,132 | 3,924,835 | 5,566,022 | 49.78% | 29.49% | 6594 |
| Inner Mongolia | 482,679 | 486,502 | 2,322,017 | 3,291,198 | 49.80% | 29.45% | 9822 |
| Liaoning | 657,653 | 668,950 | 3,301,296 | 4,627,899 | 49.57% | 28.67% | 8261 |
| Jilin | 351,694 | 359,225 | 2,049,584 | 2,760,504 | 49.47% | 25.75% | 6293 |
| Heilongjiang | 378,746 | 390,003 | 2,457,871 | 3,226,620 | 49.27% | 23.83% | 5211 |
| Shanghai | 391,860 | 403,306 | 1,578,797 | 2,373,963 | 49.28% | 33.50% | 22,667 |
| Jiangsu | 1,744,008 | 1,793,013 | 9,073,321 | 12,610,342 | 49.31% | 28.05% | 17,718 |
| Zhejiang | 1,350,393 | 1,371,542 | 5,730,292 | 8,452,227 | 49.61% | 32.20% | 15,607 |
| Anhui | 1,658,485 | 1,678,288 | 8,118,468 | 11,455,241 | 49.70% | 29.13% | 8406 |
| Fujian | 1,164,949 | 1,193,898 | 5,475,881 | 7,834,729 | 49.39% | 30.11% | 15,484 |
| Jiangxi | 1,238,615 | 1,254,197 | 7,216,141 | 9,708,953 | 49.69% | 25.68% | 7653 |
| Shandong | 2,976,718 | 3,111,694 | 12,540,322 | 18,628,734 | 48.89% | 32.68% | 10,152 |
| Henan | 2,843,483 | 2,913,877 | 16,773,572 | 22,530,932 | 49.39% | 25.55% | 8081 |
| Hubei | 1,368,695 | 1,380,565 | 6,432,827 | 9,182,087 | 49.78% | 29.94% | 11,105 |
| Hunan | 1,693,119 | 1,725,210 | 9,268,434 | 12,686,764 | 49.53% | 26.94% | 8359 |
| Guangdong | 3,663,680 | 3,683,541 | 15,757,287 | 23,104,508 | 49.86% | 31.80% | 13,672 |
| Guangxi | 1,642,345 | 1,658,134 | 8,258,333 | 11,558,812 | 49.76% | 28.55% | 6219 |
| Hainan | 288,359 | 288,677 | 1,384,674 | 1,961,710 | 49.97% | 29.41% | 8214 |
| Chongqing | 698,248 | 707,656 | 3,572,746 | 4,978,650 | 49.67% | 28.24% | 10,977 |
| Sichuan | 1,861,381 | 1,884,495 | 9,407,955 | 13,153,831 | 49.69% | 28.48% | 8030 |
| Guizhou | 1,379,285 | 1,363,587 | 6,236,476 | 8,979,348 | 50.29% | 30.55% | 6722 |
| Yunnan | 1,406,565 | 1,401,411 | 6,171,909 | 8,979,885 | 50.09% | 31.27% | 6940 |
| Tibet | 140,100 | 139,064 | 590,507 | 869,671 | 50.19% | 32.10% | 7079 |
| Shaanxi | 1,033,760 | 1,048,041 | 4,593,907 | 6,675,709 | 49.66% | 31.18% | 9648 |
| Gansu | 738,882 | 741,632 | 3,241,524 | 4,722,038 | 49.91% | 31.35% | 4776 |
| Qinghai | 179,776 | 178,288 | 841,265 | 1,199,329 | 50.21% | 29.86% | 7031 |
| Ningxia | 224,351 | 223,518 | 978,621 | 1,426,491 | 50.09% | 31.40% | 7848 |
| Xinjiang | 610,724 | 635,531 | 4,470,539 | 5,716,794 | 49.00% | 21.80% | 7858 |

**Table S2. Proportion of urban population in China**

|  | **Proportion of 6 months–2 years children in urban areas** | **Proportion of 3–4 years children in urban areas** | **Proportion of 5–14 years children in urban areas** |
| --- | --- | --- | --- |
| Nation | 63.69% | 64.87% | 60.00% |
| Beijing | 87.46% | 87.76% | 89.92% |
| Tianjin | 85.36% | 85.71% | 81.54% |
| Hebei | 62.31% | 63.34% | 57.06% |
| Shanxi | 67.91% | 69.40% | 67.44% |
| Inner Mongolia | 76.65% | 77.43% | 71.73% |
| Liaoning | 79.76% | 80.51% | 72.73% |
| Jilin | 72.49% | 73.15% | 62.44% |
| Heilongjiang | 71.86% | 72.46% | 65.36% |
| Shanghai | 92.97% | 93.24% | 94.29% |
| Jiangsu | 77.64% | 78.38% | 74.66% |
| Zhejiang | 75.39% | 76.32% | 74.98% |
| Anhui | 58.70% | 60.02% | 55.19% |
| Fujian | 69.87% | 70.91% | 69.11% |
| Jiangxi | 58.52% | 60.25% | 58.25% |
| Shandong | 67.57% | 68.76% | 63.35% |
| Henan | 53.19% | 54.53% | 50.06% |
| Hubei | 62.75% | 64.11% | 61.56% |
| Hunan | 58.16% | 59.82% | 56.13% |
| Guangdong | 69.40% | 70.36% | 67.24% |
| Guangxi | 52.74% | 53.87% | 49.39% |
| Hainan | 61.07% | 62.27% | 58.22% |
| Chongqing | 74.27% | 75.47% | 68.59% |
| Sichuan | 58.33% | 59.69% | 54.08% |
| Guizhou | 52.40% | 53.72% | 49.43% |
| Yunnan | 49.25% | 50.46% | 43.52% |
| Tibet | 23.89% | 25.32% | 25.76% |
| Shaanxi | 64.39% | 65.77% | 63.86% |
| Gansu | 53.06% | 54.43% | 51.18% |
| Qinghai | 52.73% | 54.03% | 52.58% |
| Ningxia | 63.83% | 64.83% | 62.70% |
| Xinjiang | 55.09% | 54.91% | 42.89% |

**eMethods 1**

In this study, children with underlying medical conditions were defined as high-risk group. The medical conditions could increase risk of severe influenza disease or death according to world health organization guidelines. The risk conditions were provided in Table S3. The method of estimating high risk rate came from Clark et al study.^1^ Data on the prevalence of underlying conditions in 2019 were extracted by under age (5 years and 5–14 years) and cause using the Global Burden of Diseases, Risk Factors, and Injuries Study (GBD) results tool (<https://ghdx.healthdata.org/gbd-results-tool>).

We followed the medical conditions provided as the cause of illness. However, definition of some diseases was not same in GBD and influenza guideline. We searched the disease in each condition category one by one. For example, in WHO influenza guidelines, chronic renal disease included chronic renal failure, nephrotic syndrome, and renal transplantation, which are different from that in GBD tool. We searched the prevalence of chronic kidney disease due to diabetes mellitus type 1, chronic kidney disease due to diabetes mellitus type 2, chronic kidney disease due to hypertension, chronic kidney disease due to glomerulonephritis, and chronic kidney disease due to other and unspecified causes in GBD tool. We provided search item in tool in Table S3. Additionally, the prevalence of obesity among children was not reported in China. We borrowed data from one national study in China.^2^

Moreover, we also estimated prevalence of people who have more than one disease to avoid an overestimate of high-risk rate. The method was also provided in Clark et al study.^1^ The ratio between the observed and expected percentage of individuals with at least one condition was derived from one study in China.^3^ The ratio among children under 5 years and ≥5 years was set to 1.10 and 1.00 respectively.

**Reference**

[1] Clark A, Jit M, Warren-Gash C, et al. Global, regional, and national estimates of the population at increased risk of severe COVID-19 due to underlying health conditions in 2020: a modelling study. Lancet Glob Health. 2020;8(8):e1003-e1017.

[2] Pan XF, Wang L, Pan A. Epidemiology and determinants of obesity in China [published correction appears in Lancet Diabetes Endocrinol. 2021 Jul;9(7):e2]. Lancet Diabetes Endocrinol. 2021;9(6):373-392.

[3] Wang HH, Wang JJ, Wong SY, et al. Epidemiology of multimorbidity in China and implications for the healthcare system: cross-sectional survey among 162,464 community household residents in southern China. BMC Med 2014; 12: 188.

**Table S3. Risk conditions definition and estimated high-risk proportions of age-specific children**

| **Risk condition** | **Examples, definitions:** | **GBD search item** | **Value (Range)** |
| --- | --- | --- | --- |
| Chronic respiratory disease | Chronic obstructive pulmonary disease (COPD), including chronic bronchitis and emphysema, bronchiectasis, cystic fibrosis, interstitial lung fibrosis, pneumoconiosis, and bronchopulmonary dysplasia (BPD). Asthma is not included in this group and should be reported separately. | Chronic obstructive pulmonary disease; Silicosis; Asbestosis; Coal workers pneumoconiosis; Other pneumoconiosis; Interstitial lung disease and pulmonary sarcoidosis; Other Chronic respiratory disease | < 5 years: 0.05% (0.03%–0.08%)  5–14 years: 0.17% (0.12%–0.20%) |
| Asthma | For example, significant asthma would be that which requires continuous or repeated use of bronchodilators, inhaled or systemic corticosteroids, or that with previous exacerbation requiring hospital admission. | Asthma | < 5 years: 3.70% (2.04%–6.16%)  5–14 years: 4.26% (2.68%–6.70%) |
| Diabetes | Type 1 diabetes  Type 2 diabetes requiring insulin or oral hypoglycemic drugs | Type 1 diabetes  Type 2 diabetes | < 5 years: 0.01% (0.00%–0.01%)  5–14 years: 0.06% (0.04%–0.09%) |
| Chronic cardiac disease | Conditions that require regular medications or follow-up, including  Congenital heart disease  Cardiomyopathy as the result of prolonged hypertension (hypertension alone in the absence of associated heart disease is not considered a risk factor for severe outcome)  Chronic heart failure  Ischaemic heart disease | Rheumatic heart disease; Ischemic heart disease; Ischemic stroke; Intracerebral haemorrhage; Subarachnoid haemorrhage; Hypertensive heart disease; Non-rheumatic calcific aortic valve disease; Non-rheumatic degenerative mitral valve disease; Other non-rheumatic valve diseases; Myocarditis; Alcoholic cardiomyopathy; Other cardiomyopathy; Atrial fibrillation and flutter; Aortic aneurysm; Peripheral artery disease; Endocarditis; Other cardiovascular and circulatory diseases; Congenital heart anomalies | < 5 years: 0.73% (0.57%–0.97%)  5–14 years: 0.78% (0.59%–1.02%) |
| Chronic renal disease | Chronic renal failure  Nephrotic syndrome  Renal transplantation | Chronic kidney disease due to diabetes mellitus type 1; chronic kidney disease due to diabetes mellitus type 2; chronic kidney disease due to hypertension; chronic kidney disease due to glomerulonephritis; chronic kidney disease due to other and unspecified causes | < 5 years: 0.28% (0.22%–0.35%)  5–14 years: 0.54% (0.45%–0.65%) |
| Chronic liver disease | Cirrhosis  Biliary atresia  Chronic hepatitis | Cirrhosis and other chronic liver diseases due to hepatitis B; Cirrhosis and other chronic liver diseases due to hepatitis C; Cirrhosis and other chronic liver diseases due to alcohol use; Cirrhosis and other chronic liver diseases due to other causes; Cirrhosis and other chronic liver diseases due to NAFLD | < 5 years: 1.24% (0.98%–1.55%)  5–14 years: 2.10% (1.76%–2.49%) |
| Chronic neurological disease | Stroke with persistent neurological deficit  Neuromuscular diseases associated with impaired respiratory function or risk of aspiration, such as cerebral palsy or myasthenia gravis  Severe developmental disorder in children | Alzheimer's disease and other dementias; Parkinson's disease; Multiple sclerosis; Motor neuron disease; Other neurological disorders; Idiopathic developmental intellectual disability; Down syndrome; Neural tube defects | < 5 years: 0.54% (0.18%–0.91%)  5–14 years: 0.63% (0.28%–0.98%) |
| Chronic haematological disorder  Immune compromise (as a result of disease or treatment) | Thalessemia major  Aplastic anemia  Immunodeficiencies related to use of immunosuppressive drugs (e.g. chemotherapy or drugs used to suppress transplant rejection) or systemic steroids  Asplenia or splenic dysfunction (e.g. with sickle cell anemia)  Human Immunodeficiency Virus infection or Acquired Immune Deficiency Syndrome (HIV/AIDS) | HIV/AIDS - Drug-susceptible Tuberculosis; HIV/AIDS - Multidrug-resistant Tuberculosis without extensive drug resistance; HIV/AIDS - Extensively drug-resistant Tuberculosis; HIV/AIDS resulting in other diseases | < 5 years: 0.00% (0.00%–0.01%)  5–14 years: 0.00% (0.00%–0.01%) |
| Chronic haematological disorder  Immune compromise (as a result of disease or treatment) | Sickle cell disease | Sickle cell disorders | < 5 years: 0.00% (0.00%–0.00%)  5–14 years: 0.00% (0.00%–0.00%) |
| Obesity parameter, Body Mass Index (BMI) | BMI is calculated as body weight in kilograms divided by the square of the height in meters (kg/m2). WHO defines obesity as a BMI of > 30 kg/m^2^. A commonly used definition for extreme or morbid obesity is a BMI > 40 kg/m^2^ | None | < 5 years: 3.6% (2.52%–14.40%)  5–14 years: 7.9% (5.53%–10.27%) |
| Tuberculosis | History of or current symptomatic tuberculosis requiring treatment. | Drug-susceptible tuberculosis; Multidrug-resistant tuberculosis without extensive drug resistance; Extensively drug-resistant tuberculosis | < 5 years: 0.04% (0.03%–0.06%)  5–14 years: 0.02% (0.01%–0.03%) |

**eMethods 2**

According to the Chinese surveillance program, we used the multiplier model method described by Reed et al ^1^ and made further improvements based on the revised model offered by Wu et al. ^2^ The true total number of infections was estimated by dividing the number of positive specimens by nine proportions in this multiplier modelling.^3^

For national perspective, we obtained the number of positive specimens from the influenza weekly report published by the Chinese National Influenza Center (<http://www.chinaivdc.cn/cnic/>). The influenza weekly report did not provide the percentage of positive specimens according to age, so we used the parameter from an epidemiologic study conducted in Jiangsu province, China, between 2010 and 2014.^4^ The proportions were 41.02%, 26.43%, 31.35%, and 7.92% in the 0–4 years, 5–18 years, 19–59 years, and ≥60 years age groups, respectively. We calculated number of positive specimens among 0-4y and 5-14y and then estimated the number of symptomatic influenza illnesses in 2019–2020 influenza season. The incidence of symptomatic illnesses per 1000 persons was estimated by dividing the number of values by the size of the population nationally.

The influenza weekly report also did not provide the number of province-specific positive specimens. We searched the studies or documents on China National Knowledge Infrastructure and official health websites to obtain number of positive specimens in 2019–2020 influenza season in various provinces. Additionally, there were a few provinces which did not report related data. We calculated the symptomatic rate in the cities in these provinces. For example, the number of positive influenza specimens in Tianjin was not provided in 2019–2020 season. The incidence of symptomatic cases was calculated in Binghai district in Tianjin was calculated to as the proxy of symptomatic rate in Tianjin city. The incidence of symptomatic cases per 1000 individuals was estimated by dividing the number of values by the size of the population at provincial level.

**Table S4 Number of positive specimens and estimated influenza symptomatic rate**

|  | **Number of positive specimens, < 5 years children** | **Number of positive specimens, 5–14 years children** | **Symptomatic rate (per 1000 individuals), < 5 years children (95% uncertainty range)** | **Symptomatic rate (per 1000 individuals), 5**–**14 years children (95% uncertainty range)** |
| --- | --- | --- | --- | --- |
| Nation | 21,522 | 13,867 | 58.63 (36.25–97.57) | 15.47 (9.62–25.64) |
| Beijing ^5^ | 454 | 571 | 116.17 (70.86–198.77) | 83.44 (49.97–141.85) |
| Tianjin (Binghai district) ^6^ | 10 | 26 | 74.99 (45.61–130.57) | 92.20 (55.81–156.56) |
| Hebei ^7^ | 637 | 552 | 28.01 (16.76–47.61) | 12.89 (7.75–22.11) |
| Shanxi ^8^ | 332 | 619 | 50.23 (30.52–85.84) | 32.43 (19.51–55.30) |
| Inner Mongolia ^9,10^ | 111 | 220 | 26.48 (15.86–44.92) | 20.35 (12.23–35.14) |
| Liaoning ^11^ | 720 | 591 | 127.70 (77.22–220.98) | 39.03 (23.35–66.95) |
| Jilin ^12,13^ | 112 | 171 | 27.98 (16.84–48.34) | 17.50 (10.46–30.16) |
| Heilongjiang ^14,15^ | 165 | 213 | 40.65 (24.50–70.07) | 18.56 (11.22–31.74) |
| Shanghai (Songjiang district) ^16^ | 85 | 103 | 259.08 (169.66–398.54) | 219.11 (139.51–348.09) |
| Jiangsu (Huai’an city) ^17,18^ | 115 | 105 | 100.39 (66.30–157.38) | 44.29 (29.14–68.48) |
| Zhejiang (Huzhou city) ^19,20^ | 101 | 79 | 207.11 (124.11–357.46) | 67.64 (40.76–116.56) |
| Anhui (Anqing city) ^21^ | 74 | 175 | 54.81 (36.13–85.27) | 55.41 (36.95–87.02) |
| Fujian (Nanping city) ^22^ | 115 | 69 | 6.55 (4.31–10.15) | 37.06 (24.52–58.20) |
| Jiangxi (Nanchang city) ^23^ | 20 | 36 | 10.88 (7.14–17.03) | 8.94 (5.88–13.79) |
| Shandong (Dezhou city) ^24^ | 52 | 110 | 35.07 (20.93–60.45) | 33.16 (19.80–57.26) |
| Henan ^25,26^ | 892 | 1718 | 27.47 (16.43–47.43) | 25.53 (15.44–43.50) |
| Hubei ^27,28^ | 1646 | 509 | 107.62 (71.79–168.10) | 16.02 (10.50–24.89) |
| Hunan ^29,30^ | 524 | 330 | 24.44 (16.27–38.09) | 7.08 (4.66–10.96) |
| Guangdong (Foshan city) ^31^ | 293 | 343 | 158.23 (103.83–245.89) | 70.36 (46.40–109.02) |
| Guangxi (Nanning city) ^32^ | 131 | 107 | 52.63 (35.06–82.26) | 18.69 (12.40–29.24) |
| Hainan ^33^ | 413 | 373 | 137.35 (90.49–212.83) | 53.86 (35.50–84.12) |
| Chongqing ^34,35^ | 196 | 603 | 24.98 (16.35–38.95) | 29.97 (19.78–46.69) |
| Sichuan ^36^ | 2911 | 2867 | 148.06 (97.45–231.54) | 55.11 (36.70–86.01) |
| Guizhou ^37,38^ | 1147 | 725 | 108.20 (71.65–170.23) | 21.28 (14.02–33.33) |
| Yunnan ^39^ | 502 | 427 | 43.17 (28.54–67.26) | 13.13 (8.67–20.44) |
| Tibet ^40^ | 72 | 48 | 55.09 (33.11–94.34) | 15.18 (9.15–26.05) |
| Shaanxi (Hanzhong city) ^41^ | 50 | 112 | 73.87 (44.58–124.39) | 66.13 (39.45–111.28) |
| Gansu ^42^ | 711 | 492 | 137.19 (82.78–233.39) | 32.41 (19.71–56.03) |
| Qinghai (Xining city) ^43^ | 50 | 86 | 84.39 (50.59–144.45) | 53.18 (31.90–90.81) |
| Ningxia ^44,45^ | 185 | 251 | 96.66 (58.29–167.78) | 48.25 (28.90–82.78) |
| Xinjiang ^46,47^ | 339 | 493 | 38.83 (23.38–67.10) | 24.29 (14.64–41.54) |

**Reference**

[1] Reed C, Angulo FJ, Swerdlow DL, et al. Estimates of the prevalence of pandemic (H1N1) 2009, United States, April-July 2009. Emerg Infect Dis. 2009;15(12):2004-2007.

[2] Wu S, VAN Asten L, Wang L, et al. Estimated incidence and number of outpatient visits for seasonal influenza in 2015-2016 in Beijing, China. Epidemiol Infect. 2017;145(16):3334-3344.

[3] Wang Q, Yang L, Liu C, Jin H, Lin L. Estimated Incidence of Seasonal Influenza in China From 2010 to 2020 Using a Multiplier Model. JAMA Netw Open. 2022;5(4):e227423.

[4] Liao Y, Xue S, Xie Y, et al. Characterization of influenza seasonality in China, 2010-2018: Implications for seasonal influenza vaccination timing. Influenza Other Respir Viruses. 2022;16(6):1161-1171.

[5] Wu S, VAN Asten L, Wang L, et al. Estimated incidence and number of outpatient visits for seasonal influenza in 2015-2016 in Beijing, China. Epidemiol Infect. 2017;145(16):3334-3344.

[6] Wang Y, Wang F, Yang Y, Wang Y, Jiao LY. Analysis of etiological characteristics of influenza in Binhai New Area of Tianjin from 2014 to 2017. Chin J Health Lab Tec. 2018,28(17):2165-2167+2170.

[7] Li Y, Han GY, Jiang CX, Liu YF, Liu LF, Qi SX. Sentinel surveillance for etiology of influenza-like illness among 0-14-year-old children of Hebei province during the 2015-2020 influenza seasons. CHINESE JOURNAL OF VACCINES AND IMMUNIZATION. 2021,27(04):410-413.

[8] Liu L, Ren BZ, Zhang RJ, Zhao JY, Zhao R, Wang J. Analysis on influenza pathogenic surveillance，Shanxi province, 2019-2020. Prev Med Trib. 2020,26(09):645-647+651.

[9] Guo YH, Guo WD, Yue H. Epidemiology and Etiology Autonomous of Influenza in Inner Mongolia Region in 2018. Inner Mongolia Med J. 2019,51(08):909-911.

[10] Wang HX. Epidemiology and etiological study of influenza in the Inner Mongolia Region from 2015 to 2017. Inner Mongolia Medical University, 2018.

[11] Wang LL, Sun HB, Sun BH, et al. Analysis of influenza surveillance results in Liaoning Province from April 2017 to March 2018. CHINA MODERN MEDICINE. 2019,26(09):174-177.

[12] Liu HM, Li J, Hou CC, Li X, Yang XD, Wu DL. Etiological surveillance and analysis of influenza in ,/ilin Province from 2019 to 2020. Chin J Lab Diagn. 2020,24(10):1583-1586.

[13] Yang CC, Deng LQ, Luan B, Lu XR, Sun PH, Bai B. Etiological surveillance of influenza in Jilin Province, 2012-2014. Pract Prev Med. 2016,23(10):1164-1166.

[14] Liu L, Wang B, Zhou D, Ma YJ, Zhang WN. Analysis of Nucleic Acid Detection Results of Influenza Virus in Jiamusi from 2009 to 2020. CHINESE PRIMARY HEALTH CARE. 2021,35(10):68-70.

[15] Zhou GE, Shu C, Xu J, Shi X, Leng Y. Quality assessment of influenza surveillance in Heilongjiang, 2018—2019. Chin J of PHM. 2020,36(03):404-406+410.

[16] Yao XQ, Zhang QH, Wu JJ, Liu L, Kong YY. Influenza surveillance among children in SongJiang District of Shanghai, 2014-2018. Shanghai Journal of Preventive Medicine. 2021,33(01):56-60.

[17] Tang L, He F, Yang PF, Li SZ. Analysis of influenza epidemic surveillance in Huai' an City in 2017-2019. J Pub Health Prev Med. 2021,32(04):75-79.

[18] Zi HR. Influenza Surveillance and Molecular Epidemiology of Influenza A /H1N1 (09pdm) viruses, Jiangsu province, 2010-2014. Southeast University (master dissertation).2015.

[19] Li YF, Fu XF, Qi YP, et al. Analysis of influenza surveillance in sentinel hospitals in Jiaxing, Zhejiang Province in the epidemic year 2015-2018. International Journal of Epidemiology and Infectious. 2020,47(02):116-119.

[20] Liu Y, Shen JY, Wen D, Liu GT. Influenza surveillance in Huzhou during 2011-2017. Shanghai Journal of Preventive Medicine. 2021,33(02):115-119.

[21] Wu ZC, Xu SQ, Li XX, et al. Analysis of influenza surveillance results in Anqing from 2018 to 2019. Pract Prev Med. 2019,26(12):1501-1504.

[22] He Y, Liao YH, Guo ZM, et al. Analysis of influenza surveillance results in Longyan City from 2014 to 2018. Strait J Prev Med. 2020,26(05):34-36.

[23] He FL, Xia W, Zhou XF, Fan GY, Wu JW, Ni XS. Analysis of pathogenic surveillance results for influenza from sentinel hospitals in Nanchang during 2011-2015. Chin J Health Lab Tec. 2016,26(18):2679-2681+2685.

[24] Wang GQ, Cao J, Wang DM, Cui YB, Xu BS, Dong J. Analysis on epidemiological characteristics and etiology of influenza, Dezhou city, 2009-2019. Prev Med Trib. 2020,26(06):434-439.

[25] Xia H, Jia GH, Seng MH et al. Surveillance results of influenza-like illness in Xuchang, Henan, 2016-2020. Henan J Prev Med. 2021,32(07):525-527.

[26] Yu Y, Ji YF, Zhao S, et al. Surveillance and Analysis of influenza in Henan province in 2017-2018. Henan J Prev Med. 2020,31(09):674-676.

[27] Wang P, Yang XB, Kong DG, Wang Y. Analysis of influenza surveillance in Wuhan, 2012一2017. Modern Preventive Medicine. 2018,45(01):141-144.

[28] Liu LL, Han S, Yu X, Li X, Ye GJ. Epidemiology and etiology of influenza in Hubei province, 2016-2019. Disease Surveillance. 2020,35(12):1105-1109.

[29] Huang YW, Zhang H, Zhang SY, et al. Analysis of pathogenic detection results of influenza sentinel surveillance in Hunan Province from 2014 to 2017. Chin J Dis Control Prev. 2018,22(01):42-45.

[30] Huang CY, Zhang HJ, Gao LD, et al. Influenza survelliance in Hunan Province, 2007一2016. Pract Prev Med. 2018,25(08):897-901.

[31] Zou L, Xie DD, Wu MX, Li Y. Surveillance of influenza in children at sentinel hospitals in Foshan City, 2012-2019. Pract Prev Med. 2021,28(04):450-453.

[32] Zhan XJ, Luo MF, Nong H, Pei JX, Yang C, Qin JQ. Pathogenic surveillance results of influenza in Nanning, 2014-2017. Modern Preventive Medicine. 2018,45(17):3191-3194.

[33] Cui L, Feng FL, Wang RM, et al. Surveillance analysis of influenza in Hainan, 2016-2020. Modern Preventive Medicine. 2020,47(16):3028-3032.

[34] Luo X, Xiao L, Liu LJ. Analysis on the sentinel surveillance of influenza in Ba’nan district of Chongqing in 2009-2011. Chinese Journal of Health Laboratory technology. 2013,23(03):740-742+745.

[35] Su K. The study of epidemiologic characteristics and forecasting of influenza in Chongqing, China. Army Medical University,2020.

[36] Zhou LJ, Wu Y, Cheng XW, et al. Influenza surveillance analysis in Sichuan from 2015 to 2019. Modern Preventive Medicine. 2021,48(10):1887-1890+1905.

[37] Wan YH, Zhen QN, Ren LJ, et al. Analysis of etiologial characteristics of influenza in Guizhou province during 2015 to 2016. Modern Preventive Medicine. 2017,44(02):323-327.

[38] Lei MY, Wan YH, Zhuang L, et al. Surveillance on influenza一like cases in Guizhou，2012一2019. Modern Preventive Medicine. 2020,47(15):2835-2838+2876.

[39] Zhang LF, Zhao XN, Li D et al. Analysis on influenza surveillance data from 2016 to 2018 in Yunnan province of China. Chin J Viral Dis. 2021,11(01):6-10.

[40] Zha XSM, Cai Z, Ci RDZ, Bai MCW, Da Z, Ci RDJ. Analysis of influenza epidemic characteristics in Tibet Autonomous Region from 2013 to 2016. Tibetan medicine. 2018,39(04):79-80.

[41] Zhang JD, Gao J, Han YF, Deng T, Li SL, Tang WJ. Results of Influenza Surveillance in Hanzhong City From 2016 to 2019. J Prev Med Inf. 2021,37(01):33-37.

[42] Zhang H, Li HY, Xu CB, et al. Analysis of influenza virus surveillance results in Gansu Province from 2010 to 2018. Chinese Journal of Experimental and Clinical Virology. 2020,34(3):276-279.

[43] Wu J, Sun YL, Wang Y, Zhang R. Influenza sentinel hospitals surveillance in Xining City, 2014一2018. Modern Preventive Medicine. 2020,47(03):405-408.

[44] Wen QF, Sun XQ, Yuan F et al. Etiology surveillance and analysis of influenza in Ningxia during 2009一2013. Chin J Health Lab Tec. 2015,25(5):705-707.

[45] Wen QF, Sun XQ, Ma XY et al. Analysis of influenza surveillance results in Ningxia from 2016 to 2018. Ningxia Med J. 2019,41(10):884-886.

[46] Li N, Ma HMT, Gu ZG, Zhao J, Zhang X, Chen Y. Analysis on epidemiological characteristics and antigenicity of influenza in Xinjiang from 2018-2019. Occup and Health 2021,37(05):620-623.

[47] Zhao J, Chen Y, Zhang X, Li N, Liu HB, Ma HMT. Analysis of detection data of influenza network laboratory in Xinjiang from 2017 to 2020. Bull Dis Control Prev. 2021,36(03):10-13.

**eMethods 3**

The provincial-specific influenza-associated mortality was obtained from Li et al study.^1^ Meanwhile, we adjusted the data extracted from this study. This study estimated mortality in China between 2010 and 2015. The influenza-associated excess respiratory mortality was associated with symptomatic influenza rate. Wang et al reported that the number of symptomatic influenza rate in 2019-2020 season was considerably higher that between 2010 and 2015, almost 3.81 times.^2^ The mortality was adjusted through multiplying by the by the multiple relationship between symptomatic influenza rates.

According to Yang et al method,^3^ the case fatality ratio of hospitalization in the decision model was followed:

$$Case fatality ratio of hospitalization=$$

$$\frac{influenza-associated mortality}{proportion of hospitalization among seeking healthcare cases * symptomatic influenza rate * proportion of cases seeking healthcare among symptomatic cases}$$

In one-way sensitive analysis, these four parameters were fully dependent. However, the case fatality ratio of hospitalization was dependent on the four parameters.

[1] Li L, Liu Y, Wu P, et al. Influenza-associated excess respiratory mortality in China, 2010-15: a population-based study. Lancet Public Health. 2019;4(9):e473-e481. doi:10.1016/S2468-2667(19)30163-X

[2] Wang Q, Yang L, Liu C, Jin H, Lin L. Estimated Incidence of Seasonal Influenza in China From 2010 to 2020 Using a Multiplier Model. JAMA Netw Open. 2022;5(4):e227423.

[3] Yang J, Atkins KE, Feng L, et al. Cost-effectiveness of introducing national seasonal influenza vaccination for adults aged 60 years and above in mainland China: a modelling analysis. BMC Med. 2020;18(1):90. Published 2020 Apr 14. doi:10.1186/s12916-020-01545-6

**Table S5.** **Influenza-associated excess respiratory mortality at national-and-provincial level**

|  | **Influenza-associated excess respiratory mortality (per 100,000 individuals)，6 months**–**14 years children (95% confidence intervals)** |
| --- | --- |
| Nation | 5.72 (4.19–7.24) |
| Beijing | 12.46 (4.72–19.96) |
| Tianjin | 4.53 (0.00–9.11) |
| Hebei | 8.42 (0.00–17.98) |
| Shanxi | 3.96 (0.00–14.14) |
| Inner Mongolia | 10.52 (0.00–22.06) |
| Liaoning | 2.29 (0.00–6.36) |
| Jilin | 4.04 (1.18–6.71) |
| Heilongjiang | 0.99 (0.00–3.66) |
| Shanghai | 31.24 (22.17–39.97) |
| Jiangsu | 0.38 (0.00–5.94) |
| Zhejiang | 4.34 (0.00–8.38) |
| Anhui | 1.79 (0.00–6.06) |
| Fujian | 2.29 (0.00–8.99) |
| Jiangxi | 0.88 (0.00–9.30) |
| Shandong | 5.26 (0.50–9.91) |
| Henan | 0.91 (0.00–3.81) |
| Hubei | 3.35 (0.00–7.05) |
| Hunan | 2.06 (0.00–9.30) |
| Guangdong | 6.93 (0.00–15.85) |
| Guangxi | 10.86 (0.00–22.94) |
| Hainan | 11.89 (2.86–20.76) |
| Chongqing | 4.38 (0.00–15.13) |
| Sichuan | 2.97 (0.00–12.46) |
| Guizhou | 10.67 (0.00–23.66) |
| Yunnan | 11.96 (0.50–23.16) |
| Tibet | 18.44 (8.31–28.31) |
| Shaanxi | 4.53 (0.00–8.69) |
| Gansu | 6.21 (0.00–16.50) |
| Qinghai | 14.94 (4.23–25.45) |
| Ningxia | 14.94 (4.04–25.34) |
| Xinjiang | 14.82 (4.04–25.41) |

**Table S6. Prices of vaccines**

| **Vaccine type** | **Vaccine productor** | **Price (CNY)/ dose** | **Website** |
| --- | --- | --- | --- |
| **Children’s type** |  |  |  |
| Trivalent inactivated influenza vaccine | SINOVAC BIOTECH CO., LTD. | 35 | http://www.ccgp.gov.cn/cggg/dfgg/zbgg/202009/t20200916_15078640.htm |
| Trivalent inactivated influenza vaccine (Children’s type) | Shenzhen Sanofi Pasteur Bioproduct Ltd | 45 |  |
| Trivalent inactivated influenza vaccine (Children’s type) | Changchun Institute of Biological Products Co., ltd | 31 |  |
| Trivalent inactivated influenza vaccine (Children type) | Shenzhen Sanofi Pasteur Bioproduct Ltd | 29 | http://www.ccgp.gov.cn/cggg/dfgg/zbgg/201909/t20190927_13006146.htm |
| Trivalent inactivated influenza vaccine (Children type) | SINOVAC BIOTECH CO., LTD. | 25 |  |
| Trivalent inactivated influenza vaccine (Children type) | HUALAN BIOLOGICAL ENGINEERING, INC | 28 |  |
| **Adults’ type** |  |  |  |
| Trivalent inactivated influenza vaccine | Shanghai Institute of Biological Products Co., ltd | 48 | http://www.sxsyxcg.com/HomePage/ShowDetailNew.aspx?InfoId=2397 |
| Trivalent inactivated influenza vaccine | HUALAN BIOLOGICAL ENGINEERING, INC | 25 | http://www.ccgp.gov.cn/cggg/dfgg/cjgg/202009/t20200927_15149066.htm |
| Trivalent inactivated influenza vaccine | Shenzhen Sanofi Pasteur Bioproduct Ltd | 25 |  |
| Trivalent inactivated influenza vaccine | SINOVAC BIOTECH CO., LTD. | 55 | http://www.ccgp.gov.cn/cggg/dfgg/zbgg/202009/t20200916_15078640.htm |
| Trivalent inactivated influenza vaccine | HUALAN BIOLOGICAL ENGINEERING, INC | 58 |  |
| Trivalent inactivated influenza vaccine | Shenzhen Sanofi Pasteur Bioproduct Ltd | 65 |  |
| Trivalent inactivated influenza vaccine | Beijing Shouhui Pharmaceutical CO., LTD. | 128 |  |
| Trivalent inactivated influenza vaccine | Changchun Institute of Biological Products Co., Ltd. | 25 | http://www.ccgp.gov.cn/cggg/dfgg/zbgg/201908/t20190816_12698628.htm |
| Trivalent inactivated influenza vaccine | HUALAN BIOLOGICAL ENGINEERING, INC | 25 |  |
| Trivalent inactivated influenza vaccine | SINOVAC BIOTECH CO., LTD. | 24 |  |
| Trivalent inactivated influenza vaccine | Shenzhen Sanofi Pasteur Bioproduct Ltd. | 45 | http://www.ccgp.gov.cn/cggg/dfgg/zbgg/201909/t20190927_13006146.htm |
| Trivalent inactivated influenza vaccine | SINOVAC BIOTECH CO., LTD. | 42 |  |
| Trivalent inactivated influenza vaccine | HUALAN BIOLOGICAL ENGINEERING, INC | 46 |  |
| Trivalent inactivated influenza vaccine | Chengfeng BIOTECH CO., LTD. | 128 | http://www.ccgp.gov.cn/cggg/dfgg/cjgg/201909/t20190927_13006135.htm |

**Table S7. Estimated parents’ cost due to bringing child to vaccination clinics (USD)**

|  | **Mean hourly wage in urban area in 2019** | **Mean hourly wage in rural area in 2019** | **Productivity loss in urban area** | **Productivity loss in urban area** | **Transportation Cost** | **Parents’ cost due to bringing child to vaccination clinics in urban area** | **Parents’ cost due to bringing child to vaccination clinics in rural area** |
| --- | --- | --- | --- | --- | --- | --- | --- |
| Nation | 2.65 | 1.00 | 3.79 | 1.43 | 0.8 | 4.590 | 2.233 |
| Beijing | 4.62 | 1.81 | 6.61 | 2.59 | 0.8 | 7.408 | 3.388 |
| Tianjin | 2.89 | 1.55 | 4.13 | 2.22 | 0.8 | 4.926 | 3.019 |
| Hebei | 2.24 | 0.96 | 3.20 | 1.38 | 0.8 | 3.998 | 2.175 |
| Shanxi | 2.08 | 0.81 | 2.98 | 1.15 | 0.8 | 3.776 | 1.954 |
| Inner Mongolia | 2.55 | 0.96 | 3.65 | 1.37 | 0.8 | 4.449 | 2.167 |
| Liaoning | 2.49 | 1.01 | 3.56 | 1.44 | 0.8 | 4.359 | 2.241 |
| Jilin | 2.02 | 0.93 | 2.89 | 1.34 | 0.8 | 3.690 | 2.136 |
| Heilongjiang | 1.94 | 0.94 | 2.77 | 1.34 | 0.8 | 3.569 | 2.140 |
| Shanghai | 4.61 | 2.08 | 6.59 | 2.97 | 0.8 | 7.387 | 3.770 |
| Jiangsu | 3.19 | 1.42 | 4.57 | 2.03 | 0.8 | 5.368 | 2.829 |
| Zhejiang | 3.77 | 1.87 | 5.38 | 2.67 | 0.8 | 6.185 | 3.473 |
| Anhui | 2.35 | 0.96 | 3.36 | 1.38 | 0.8 | 4.159 | 2.179 |
| Fujian | 2.85 | 1.22 | 4.08 | 1.75 | 0.8 | 4.882 | 2.551 |
| Jiangxi | 2.29 | 0.99 | 3.27 | 1.41 | 0.8 | 4.070 | 2.213 |
| Shandong | 2.65 | 1.11 | 3.79 | 1.59 | 0.8 | 4.587 | 2.390 |
| Henan | 2.14 | 0.95 | 3.06 | 1.36 | 0.8 | 3.860 | 2.157 |
| Hubei | 2.35 | 1.03 | 3.36 | 1.47 | 0.8 | 4.164 | 2.267 |
| Hunan | 2.49 | 0.96 | 3.56 | 1.38 | 0.8 | 4.365 | 2.177 |
| Guangdong | 3.01 | 1.18 | 4.31 | 1.68 | 0.8 | 5.105 | 2.484 |
| Guangxi | 2.17 | 0.86 | 3.11 | 1.22 | 0.8 | 3.909 | 2.024 |
| Hainan | 2.25 | 0.95 | 3.22 | 1.35 | 0.8 | 4.023 | 2.152 |
| Chongqing | 2.37 | 0.95 | 3.39 | 1.35 | 0.8 | 4.195 | 2.154 |
| Sichuan | 2.26 | 0.92 | 3.23 | 1.31 | 0.8 | 4.035 | 2.113 |
| Guizhou | 2.15 | 0.67 | 3.08 | 0.96 | 0.8 | 3.878 | 1.762 |
| Yunnan | 2.27 | 0.74 | 3.24 | 1.06 | 0.8 | 4.042 | 1.865 |
| Tibet | 2.34 | 0.81 | 3.35 | 1.16 | 0.8 | 4.147 | 1.959 |
| Shaanxi | 2.26 | 0.77 | 3.23 | 1.10 | 0.8 | 4.030 | 1.903 |
| Gansu | 2.02 | 0.60 | 2.89 | 0.86 | 0.8 | 3.692 | 1.662 |
| Qinghai | 2.12 | 0.72 | 3.03 | 1.03 | 0.8 | 3.827 | 1.829 |
| Ningxia | 2.15 | 0.80 | 3.07 | 1.15 | 0.8 | 3.871 | 1.950 |
| Xinjiang | 2.17 | 0.82 | 3.10 | 1.17 | 0.8 | 3.902 | 1.974 |

**eMethods 4**

Yang et al used the number of seasonal influenza vaccine supply to estimate the influenza vaccination coverage.^1^ We obtained the data about supply of influenza vaccines from the website of the National Institutes for Food and Drug Control (<https://www.nmpa.gov.cn/>). The number of influenza vaccine supply was 28,645,538 in 2019-2020 season. After consulting with health staffs in CDC and checking the vaccination records in Wuxi city, China (unpublished data), about 60% of supply was administrated for pediatric population. The influenza vaccine coverage among children in China was estimated to be 6.95% (28,645,538*0.6/247,389,910).

Feng et al reported doses of influenza vaccine for sale per 1000 people in different provinces. After searching average number of people between 2004 and 2008 from National Bureau of Statistics of China, doses of influenza vaccine for sale in various provinces were obtained. Each province accounted for specific proportion of vaccine supply in China. We applied this proportion to calculate the vaccine supply in 2019-2020 season and estimated the influenza vaccine coverage in various provinces. The range of coverage rate under self-paid policy was between 0.5*base-value and 1.5*base-value in the sensitive analysis. Extrapolating from the experience in Beijing, vaccination coverage under the fully-funded policy was assumed to be 40% and range was set at 30%-50%. Given the high supply in Beijing, we directly used rate of 30% as a proxy of coverage rate under self-paid policy with the range of 20%-40%. The fully-funded coverage in Beijing was set at 50% with the range of 40%-60%.

[1] Yang J, Atkins KE, Feng L, et al. Seasonal influenza vaccination in China: Landscape of diverse regional reimbursement policy, and budget impact analysis. Vaccine. 2016;34(47):5724-5735. doi:10.1016/j.vaccine.2016.10.013

[2] Feng L, Mounts AW, Feng Y, et al. Seasonal influenza vaccine supply and target vaccinated population in China, 2004-2009. Vaccine. 2010;28(41):6778-6782. doi:10.1016/j.vaccine.2010.07.064

**Table S8. Estimated influenza vaccine coverage at national-and-provincial level**

|  | **Doses of influenza vaccine for sale per 1000 people** | **Average number of people between 2004 and 2008** | **Doses of influenza vaccine for sale between 2004 and 2008** | **Proportion of supply in various provinces** | **Total Doses supplied in 2019** | **Estimated coverage** |
| --- | --- | --- | --- | --- | --- | --- |
| National | - | - | - | 1 | 28,645,538 | 6.95% |
| Beijing | 537 | 1616 | 867,685 | 0.092 | 2,649,056 | 30.00% |
| Tianjin | 268 | 1087 | 291,209 | 0.031 | 889,066 | 29.21% |
| Hebei | 58 | 6898 | 400,084 | 0.043 | 1,221,463 | 4.96% |
| Shanxi | 71 | 3374 | 239,540 | 0.026 | 731,319 | 7.88% |
| Inner Mongolia | 42 | 2417 | 101,506 | 0.011 | 309,898 | 5.65% |
| Liaoning | 64 | 4264 | 272,922 | 0.029 | 833,234 | 10.80% |
| Jilin | 100 | 2722 | 272,240 | 0.029 | 831,153 | 18.07% |
| Heilongjiang | 48 | 3822 | 183,446 | 0.020 | 560,065 | 10.41% |
| Shanghai | 96 | 1979 | 189,965 | 0.020 | 579,966 | 14.66% |
| Jiangsu | 69 | 7650 | 527,878 | 0.056 | 1,611,619 | 7.67% |
| Zhejiang | 66 | 5071 | 334,686 | 0.036 | 1,021,802 | 7.25% |
| Anhui | 57 | 6142 | 350,105 | 0.037 | 1,068,878 | 5.60% |
| Fujian | 64 | 3584 | 229,402 | 0.024 | 700,367 | 5.36% |
| Jiangxi | 54 | 4340 | 234,382 | 0.025 | 715,571 | 4.42% |
| Shandong | 57 | 9304 | 530,339 | 0.057 | 1,619,135 | 5.21% |
| Henan | 53 | 9456 | 501,147 | 0.053 | 1,530,010 | 4.07% |
| Hubei | 71 | 5702 | 404,856 | 0.043 | 1,236,033 | 8.08% |
| Hunan | 47 | 6420 | 301,749 | 0.032 | 921,246 | 4.36% |
| Guangdong | 100 | 9460 | 946,000 | 0.101 | 2,888,154 | 7.50% |
| Guangxi | 49 | 4770 | 233,750 | 0.025 | 713,642 | 3.70% |
| Hainan | 55 | 836 | 45,991 | 0.005 | 140,411 | 4.29% |
| Chongqing | 58 | 2811 | 163,026 | 0.017 | 497,722 | 6.00% |
| Sichuan | 87 | 8147 | 708,806 | 0.076 | 2,163,998 | 9.87% |
| Guizhou | 25 | 3710 | 92,760 | 0.010 | 283,198 | 1.89% |
| Yunnan | 27 | 4481 | 120,987 | 0.013 | 369,375 | 2.47% |
| Tibet | 7 | 284 | 1991 | 0.000 | 6078 | 0.42% |
| Shaanxi | 145 | 3699 | 536,384 | 0.057 | 1,637,590 | 14.72% |
| Gansu | 56 | 2546 | 142,598 | 0.015 | 435,355 | 5.53% |
| Qinghai | 69 | 547 | 37,757 | 0.004 | 115,272 | 5.77% |
| Ningxia | 52 | 603 | 31,366 | 0.003 | 95,762 | 4.03% |
| Xinjiang | 43 | 2050 | 88,141 | 0.009 | 269,097 | 2.82% |

**Table S9. Estimated lifetime productivity loss due to influenza related-death (USD)**

|  | **Per capita disposable income in China in 2019** | **Discounted Lifetime Productivity** |
| --- | --- | --- |
| Nation | 4449 | 107,904.84 |
| Beijing | 9808 | 237,894.12 |
| Tianjin | 6138 | 148,882.20 |
| Hebei | 3715 | 90,110.88 |
| Shanxi | 3449 | 83,661.09 |
| Inner Mongolia | 4423 | 107,279.87 |
| Liaoning | 4606 | 111,721.34 |
| Jilin | 3556 | 86,241.71 |
| Heilongjiang | 3511 | 85,156.80 |
| Shanghai | 10,052 | 243,813.74 |
| Jiangsu | 5993 | 145,357.11 |
| Zhejiang | 7223 | 175,197.45 |
| Anhui | 3824 | 92,744.16 |
| Fujian | 5156 | 125,049.25 |
| Jiangxi | 3802 | 92,206.97 |
| Shandong | 4574 | 110,938.37 |
| Henan | 3460 | 83,924.42 |
| Hubei | 4099 | 99,429.18 |
| Hunan | 4007 | 97,185.63 |
| Guangdong | 5648 | 136,979.77 |
| Guangxi | 3377 | 81,905.57 |
| Hainan | 3862 | 93,671.07 |
| Chongqing | 4186 | 101,539.32 |
| Sichuan | 3576 | 86,733.26 |
| Guizhou | 2953 | 71,614.71 |
| Yunnan | 3197 | 77,530.82 |
| Tibet | 2823 | 68,468.82 |
| Shaanxi | 3571 | 86,603.35 |
| Gansu | 2771 | 67,197.82 |
| Qinghai | 3274 | 79,412.73 |
| Ningxia | 3534 | 85,711.54 |
| Xinjiang | 3344 | 81,115.59 |

**eMethods 5**

The static decision tree was developed at individual level which means probability of being vaccinated was used to figure out the individual health and economic outcomes (including cost of vaccination and cost due to influenza per person, and influenza related health utility per person) for the funded vaccination policy and self-paid vaccination policy. Then, we scale the cost and health outcomes to the population level through multiplying the health and economic outcomes per person by size of pediatric population aged 6 months -14 years at nation and provincial level. The ICER at national levels was calculated using the formula followed:

$${ICER}_{Nation}=\frac{{Cost}_{funded vaccination policy}-{Cost}_{self-paid vaccination policy}}{{Health Utility}_{funded vaccination policy}-{Health Utility}_{self-paid vaccination policy}}$$

The ICER at provincial level was calculated using the same method.

**Table S10. Different scenarios assumed**

| **Scenario** | **Description** |
| --- | --- |
| Scenario 1: indirect effect | 40% of children getting vaccinations (fully funded policy) could decrease symptomatic rate of children who don’t get vaccination by 4% comparing with 10% of children getting vaccinations (self-paid policy). |
| Scenario 2: low vaccination rate under fully-funded policy | Coverage was set to 30% |
| Scenario 3: high proportions of side effects due to vaccinations | Fever, ages < 5 years, 0.011; ages ≥ 5 years, 0.0035  Site reaction, ages < 5 years, 0.0043; ages ≥ 5 years, 0.00065  Anaphylaxis, ages < 5 years, 0.00000025; ages ≥ 5 years, 0.00000025 |
| Scenario 4: healthcare sector perspective | Only the direct medical costs for influenza patients were considered |
| Scenario 5: matched vaccine | Matched vaccine effectiveness was used |
| Scenario 6: mixed situation | indirect effect + high proportions of side effects + mismatched vaccine |

**Table S11. Comparisons of influenza burden between our findings and other studies**

|  | **Outcomes** |
| --- | --- |
| Our modelling | Incidence of hospitalization: 0.0019 (95%UR:0.0014–0.0025)  Influenza-associated mortality: 0.00014 (95%UR:0.00010–0.00019) |
| Li et al ^1^ | Excess all-cause mortality rate: 0.0001433 |
| Yu et al^2^ | Severe acute respiratory infection hospitalizations rate: 0.00115 |
| Yu et al^3^ | Influenza-associated severe acute respiratory infection hospitalization rate: 0.004 (95%CI: 0.002–0.005) |

[1] Li J, Chen Y, Wang X, Yu H. Influenza-associated disease burden in mainland China: a systematic review and meta-analysis. Sci Rep. 2021;11(1):2886. Published 2021 Feb 3. doi:10.1038/s41598-021-82161-z

[2] Yu H, Huang J, Huai Y, et al. The substantial hospitalization burden of influenza in central China: surveillance for severe, acute respiratory infection, and influenza viruses, 2010–2012. Influenza Other Respir Viruses, 2014, 8(1): 53-65.

[3] Yu J, Zhang X, Shan W, et al. Influenza-associated hospitalization in children younger than 5 years of age in Suzhou, China, 2011-2016. Pediatr Infect Dis J, 2019, 38(5): 445-452.

**Table S12. Comparison of costs and health outcome of different indirect effect values**

| **Outcomes** | **Indirect effect (1%)** | | **Indirect effect (10%)** | |
| --- | --- | --- | --- | --- |
|  | **Fully-funded policy** | **Self-paid policy** | **Fully-funded policy** | **Self-paid policy** |
| Total influenza symptomatic cases averted (Fully-funded policy vs self-paid policy) | 1,491,650  (1,241,119–1,770,781) | | 1,910,447  (1,627,200–2,243,914) | |
| Total influenza hospitalizations averted (Fully-funded policy vs self-paid policy) | 95,051  (69,676–127,245) | | 122,868  (90,296–162,658) | |
| Total influenza death averted (Fully-funded policy vs self-paid policy) | 6716  (4792–9278) | | 8593  (6164–11,829) | |
| Total cost (USD, million) | 5884 (4572–7730) | 5784 (4178–8076) | 5564 (4343–7240) | 5788 (4165–8016) |
| Incremental cost (USD, million, Fully-funded policy vs self-paid policy) | 91 (-401–457) |  | -219 (-817–228) |  |
| Total QALYs | 247,326,804  (247,286,825–247,353,509) | 247,311,100  (247,261,148–247,344,350) | 247,331,572  (247,292,103–247,356,539) | 247,311,477  (247,259,087–247,344,977) |
| Incremental QALYs (Fully-funded policy vs self-paid policy) | 15,608  (8905–25,811) |  | 20,014  (11,327–33,334) |  |
| ICER (Fully-funded policy vs self-paid policy) | 5668  (cost-saving–40,231) | | cost-saving  (cost-saving–14,774) | |


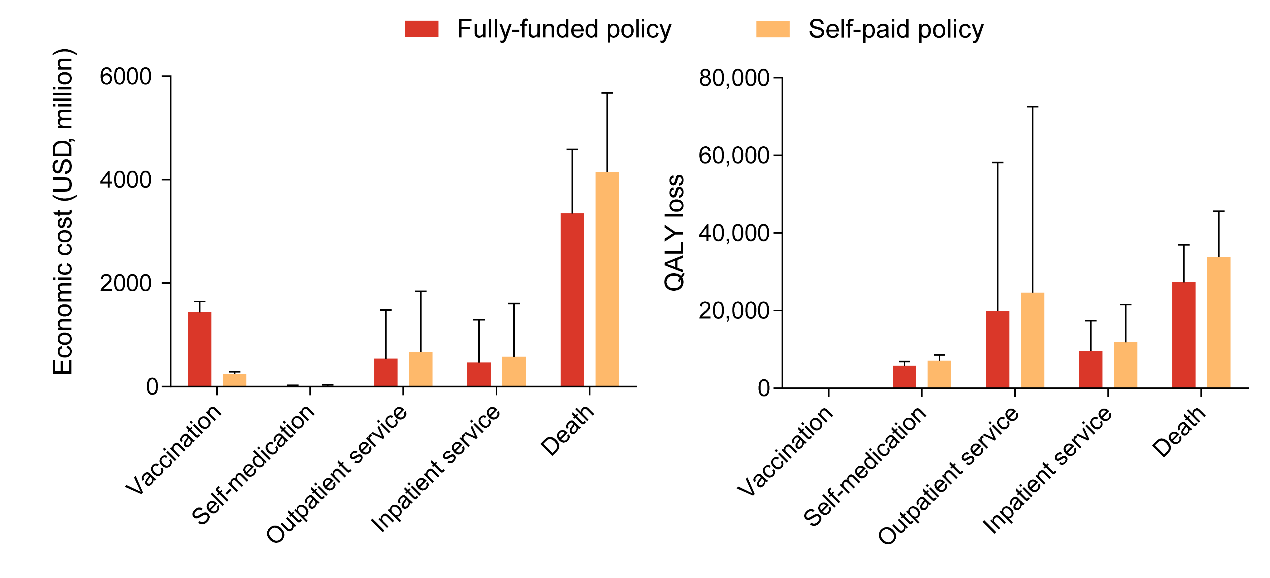


**Figure S1. Economic cost and QALY loss caused by different outcomes**

**Table S13. Comparison of costs and health outcome of vaccination strategies in various provinces**

| **Province** | **Strategy** | **Total cost (USD, million)** | **Incremental cost (USD, million, fully-funded policy vs self-paid policy)** | **Total QALYs** | **Incremental QALYs (fully-funded policy vs self-paid policy)** | **ICER (fully-funded policy vs self-paid policy)** |
| --- | --- | --- | --- | --- | --- | --- |
| Beijing | Fully-funded policy | 191  (138–284) | -17  (-32–-9) | 2,513,743  (2,512,612–2,514,471) | 217  (102–395) | cost-saving  (cost-saving–cost-saving) |
|  | Self-paid policy | 208  (147–316) | NA | 2,513,526  (2,512,226–2,514,368) | NA |  |
| Tianjin | Fully-funded policy | 57  (40–99) | -1  (-4–1) | 1,825,685  (1,824,862–1,826,200) | 66  (25–131) | cost-saving  (cost-saving–19,219) |
|  | Self-paid policy | 58  (39–103) | NA | 1,825,619  (1,824,734–1,826,175) | NA |  |
| Hebei | Fully-funded policy | 244  (203–298) | 32  (15–48) | 14,782,746  (14,781,414–14,783,620) | 630  (405–982) | 50,616  (18,481–102,288) |
|  | Self-paid policy | 212  (162–278) | NA | 14,782,113  (14,780,464–14,783,210) | NA |  |
| Shanxi | Fully-funded policy | 77  (63–104) | 14  (8–20) | 5,564,790  (5,563,732–5,565,455) | 284  (130–531) | 50,082  (19,807–121,283) |
|  | Self-paid policy | 63  (46–95) | NA | 5,564,508  (5,563,198–5,565,321) | NA |  |
| Inner Mongolia | Fully-funded policy | 85  (68–108) | 0  (-6–5) | 3,290,401  (3,290,006–3,290,679) | 197  (127–299) | 1,475  (cost-saving–34,736) |
|  | Self-paid policy | 85  (64–113) | NA | 3,290,205  (3,289,709–3,290,551) | NA |  |
| Liaoning | Fully-funded policy | 79  (57–118) | 9  (0–15) | 4,626,395  (4,624,919–4,627,345) | 316  (118–630) | 27,392  (902–88,863) |
|  | Self-paid policy | 69  (44–117) | NA | 4,626,079  (4,624,297–4,627,226) | NA |  |
| Jilin | Fully-funded policy | 33  (28–41) | 6  (4–7) | 2,760,115  (2,759,833–2,760,299) | 61  (32–108) | 91,527  (44,385–193,195) |
|  | Self-paid policy | 28  (22–36) | NA | 2,760,054  (2,759,729–2,760,266) | NA |  |
| Heilongjiang | Fully-funded policy | 28  (23–35) | 11  (9–13) | 3,226,228  (3,225,849–3,226,475) | 84  (31–165) | 131,956  (63,207–360,453) |
|  | Self-paid policy | 16  (12–25) | NA | 3,226,144  (3,225,684–3,226,444) | NA |  |
| Shanghai | Fully-funded policy | 441  (313–672) | -66  (-111–-42) | 2,370,465  (2,367,657–2372,260) | 638  (305–1157) | cost-saving  (cost-saving–cost-saving) |
|  | Self-paid policy | 508  (355–781) | NA | 2,369,824  (2,366,516–2,371,964) | NA |  |
| Jiangsu | Fully-funded policy | 284  (177–504) | 16  (-36–43) | 12,606,699  (12,602,942–12,609,050) | 847  (296–1716) | 17,353  (cost-saving–88,237) |
|  | Self-paid policy | 268  (136–538) | NA | 12,605,845  (12,601,251–12,608,749) | NA |  |
| Zhejiang | Fully-funded policy | 347  (223–589) | -21  (-74–7) | 8,447,396  (8,442,655–8,450,428) | 1044  (381–2100) | cost-saving  (cost-saving–7630) |
|  | Self-paid policy | 369  (218–661) | NA | 8,446,346  (8,440,615–8,450,052) | NA |  |
| Anhui | Fully-funded policy | 163  (120–260) | 31  (6–45) | 11,452,097  (11,448,862–11,454,121) | 778  (271–1585) | 38,567  (5753–121,503) |
|  | Self-paid policy | 132  (79–252) | NA | 11,451,321  (11,447,297–11,453,849) | NA |  |
| Fujian | Fully-funded policy | 179  (135–266) | 8  (-15–21) | 7,833,121  (7,831,875–7,833,907) | 400  (198–723) | 18,888  (cost-saving–79,124) |
|  | Self-paid policy | 171  (117–280) | NA | 7,832,719  (7,831,165–7,833,705) | NA |  |
| Jiangxi | Fully-funded policy | 76  (66–91) | 43  (36–51) | 9,708,424  (9,707,965–9,708,719) | 135  (59–257) | 319,300  (162,107–745,256) |
|  | Self-paid policy | 32  (24–49) | NA | 9,708,287  (9,707,712–9,708,660) | NA |  |
| Shandong | Fully-funded policy | 341  (270–462) | 38  (5–60) | 18,624,727  (18,621,582–18,626,707) | 1004  (500–1821) | 36,629  (4479–95,695) |
|  | Self-paid policy | 303  (216–450) | NA | 18,623,725  (18,619,748–18,626,190) | NA |  |
| Henan | Fully-funded policy | 213  (170–300) | 88  (62–110) | 22,528,023  (22,525,087–22,529,885) | 748  (268–1529) | 117,212  (51,480–335,993) |
|  | Self-paid policy | 122  (76–232) | NA | 22,527,271  (22523564–22529615) | NA |  |
| Hubei | Fully-funded policy | 152  (111–232) | 18  (0–29) | 9,179,933  (9,177,872–9,181,223) | 462  (185–918) | 37,313  (cost-saving–114,721) |
|  | Self-paid policy | 134  (85–230) | NA | 9,179,470  (9,176,992–9,181,034) | NA |  |
| Hunan | Fully-funded policy | 121  (103–144) | 44  (35–53) | 12,685,807  (12,685,059–12,686,294) | 206  (101–369) | 211,094  (111,025–450,886) |
|  | Self-paid policy | 77  (60–103) | NA | 12,685,600  (12,684,698–12,686,193) | NA |  |
| Guangdong | Fully-funded policy | 894  (618–1381) | -57  (-167–5) | 23,092,044  (23,080,672–23,099,108) | 2693  (1159–5208) | cost-saving  (cost-saving–1998) |
|  | Self-paid policy | 953  (618–1543) | NA | 23,089,366  (23,075,625–23,097,943) | NA |  |
| Guangxi | Fully-funded policy | 230  (188–287) | 14  (-3–29) | 11,555,822  (11,554,101–11,556,949) | 781  (480–1242) | 18,323  (cost-saving–50,368) |
|  | Self-paid policy | 215  (164–285) | NA | 11,555,046  (11,552,894–11,556,454) | NA |  |
| Hainan | Fully-funded policy | 59  (45–82) | -3  (-9–1) | 1,960,725  (,1959,972–1,961,215) | 253  (126–452) | cost-saving  (cost-saving–6748) |
|  | Self-paid policy | 62  (45–90) | NA | 1,960,473  (1,959,527–1,961,087) | NA |  |
| Chongqing | Fully-funded policy | 78  (62–111) | 12  (4–18) | 4,977,758  (4,976,994–4,978,231) | 218  (101–408) | 55,315  (14,983–140,763) |
|  | Self-paid policy | 65  (46–106) | NA | 4,977,540  (4,976,598–4,978,127) | NA |  |
| Sichuan | Fully-funded policy | 241  (171–378) | 19  (-13–37) | 13,148,443  (13,143,259–13,151,831) | 1170  (443–2304) | 15,389  (cost-saving–56,658) |
|  | Self-paid policy | 222  (137–389) | NA | 13,147,261  (13,140,982–13,151,376) | NA |  |
| Guizhou | Fully-funded policy | 203  (161–263) | 5  (-14–18) | 8,976,066  (,8973,770–8,977,461) | 900  (510–1550) | 5221  (cost-saving–27,518) |
|  | Self-paid policy | 198  (145–273) | NA | 8,975,163  (8,972,262–8,976,943) | NA |  |
| Yunnan | Fully-funded policy | 190  (155–237) | 9  (-6–21) | 8,977,597  (8,976,501–8,978,360) | 618  (405–930) | 13,650  (cost-saving–42,833) |
|  | Self-paid policy | 181  (138–240) | NA | 8,976,978  (8,975,584–8,977,942) | NA |  |
| Tibet | Fully-funded policy | 22  (18–28) | 0  (-2–1) | 869,373  (869,241–869,469) | 85  (56–125) | cost-saving  (cost-saving–17.624) |
|  | Self-paid policy | 22  (17–30) | NA | 869,289  (869,120–869,412) | NA |  |
| Shaanxi | Fully-funded policy | 137  (100–216) | 6  (-9–14) | 6,673,210  (6,670,913–6,674,682) | 455  (185–890) | 12,052  (cost-saving–48,335) |
|  | Self-paid policy | 131  (87–224) | NA | 6,672,757  (6,670,025–6,674,487) | NA |  |
| Gansu | Fully-funded policy | 76  (60–102) | 10  (3–15) | 4,720,262  (4,718,727–4,721,241) | 442  (195–831) | 21,269  (5064–58,132) |
|  | Self-paid policy | 66  (47–98) | NA | 4,719,818  (4,717,896–4,721,039) | NA |  |
| Qinghai | Fully-funded policy | 34  (27–45) | -1  (-4–1) | 1,198,766  (1,198,385–1,199,016) | 139  (77–236) | cost-saving  (cost-saving–7830) |
|  | Self-paid policy | 35  (26–49) | NA | 1,198,626  (1,198,151–1,198,939) | NA |  |
| Ningxia | Fully-funded policy | 45  (35–59) | -2  (-6–1) | 1,425,804  (1,425,359–1,426,105) | 178  (99–299) | cost-saving  (cost-saving–3966) |
|  | Self-paid policy | 47  (35–65) | NA | 1,425,624  (1,425,061–1,426,006) | NA |  |
| Xinjiang | Fully-funded policy | 141  (112–180) | -1  (-13–9) | 5,715,077  (5,714,197–5,715,676) | 459  (298–703) | cost-saving  (cost-saving–23,730) |
|  | Self-paid policy | 141  (106–190) | NA | 5,714,615  (5,713,511–5,715,371) | NA |  |


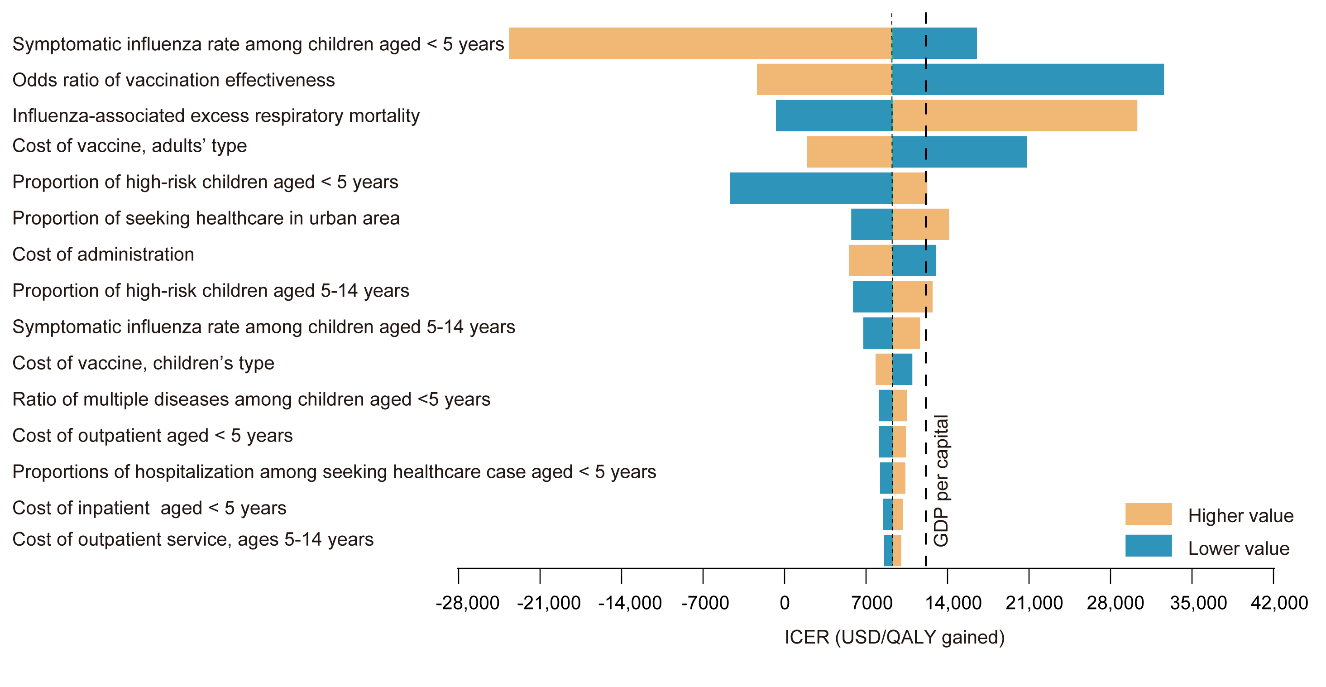


**Figure S2. One-way sensitivity analyses for the most influential model parameters on ICER (USD/QALY gained)**

**Table S14. One-way sensitivity analyses for the five most influential model parameters on ICER at the provincial level (USD/QALY gained)**

| Province | Variables | ICER | | | Provincial GDP per capita |
| --- | --- | --- | --- | --- | --- |
|  |  | Lowest value | Highest value | Spread |  |
| Beijing | Influenza-associated excess respiratory mortality | -93,739 | -29,748 | 63,991 | 23,772 |
|  | Odds ratio of vaccination effectiveness | -72,908 | -47,716 | 25,192 |  |
|  | Proportion of high-risk children aged < 5 y | -76,183 | -62,176 | 14,006 |  |
|  | QALY loss of self-medication | -71,048 | -57,833 | 13,215 |  |
|  | Duration of outpatient | -70,487 | -59,447 | 11,040 |  |
| Tianjin | Influenza-associated excess respiratory mortality | -16,299 | 25,880 | 42,180 | 13,082 |
|  | Symptomatic influenza rate among children aged 5-14 y | -12,709 | 15,186 | 27,895 |  |
|  | Odds ratio of vaccination effectiveness | -8487 | 16,747 | 25,234 |  |
|  | Proportion of seeking healthcare in urban area | -5624 | 8397 | 14,021 |  |
|  | Cost of vaccine, adults’ type | -3941 | 6029 | 9970 |  |
| Hebei | Influenza-associated excess respiratory mortality | 1449 | 256,260 | 254,811 | 6709 |
|  | Odds ratio of vaccination effectiveness | 48,471 | 108,092 | 59,621 |  |
|  | Symptomatic influenza rate among children aged 5-14 y | 45,676 | 90,911 | 45,234 |  |
|  | Cost of vaccine, adults’ type | 56,702 | 86,700 | 29,998 |  |
|  | Proportion of high-risk children aged < 5 y | 50,826 | 72,038 | 21,212 |  |
| Shanxi | Influenza-associated excess respiratory mortality | 6355 | 104,503 | 98,149 | 6619 |
|  | Odds ratio of vaccination effectiveness | 46,259 | 90,658 | 44,399 |  |
|  | Symptomatic influenza rate among children aged 5-14 y | 38,620 | 82,279 | 43,659 |  |
|  | Cost of vaccine, adults’ type | 52,745 | 74,172 | 21,427 |  |
|  | Proportion of seeking healthcare in urban area | 52,748 | 72,654 | 19,906 |  |
| Inner Mongolia | Influenza-associated excess respiratory mortality | -31,163 | 200,653 | 231,816 | 9822 |
|  | Odds ratio of vaccination effectiveness | 4326 | 50,146 | 45,819 |  |
|  | Symptomatic influenza rate among children aged 5-14 y | 3949 | 36,766 | 32,818 |  |
|  | Cost of vaccine, adults’ type | 11,713 | 32,044 | 20,331 |  |
|  | Proportion of high-risk children aged < 5 y | 6817 | 22,249 | 15,433 |  |
| Liaoning | Influenza-associated excess respiratory mortality | 17,193 | 50,494 | 33,300 | 8279 |
|  | Odds ratio of vaccination effectiveness | 26,905 | 59,477 | 32,572 |  |
|  | Symptomatic influenza rate among children aged < 5 y | 20,363 | 51,013 | 30,650 |  |
|  | Symptomatic influenza rate among children aged 5-14 y | 23,659 | 50,490 | 26,831 |  |
|  | Proportion of seeking healthcare in urban area | 29,680 | 49,638 | 19,958 |  |
| Jilin | Influenza-associated excess respiratory mortality | 64,994 | 172,211 | 107,218 | 6293 |
|  | Symptomatic influenza rate among children aged 5-14 y | 72,146 | 148,093 | 75,946 |  |
|  | Odds ratio of vaccination effectiveness | 82,398 | 150,684 | 68,286 |  |
|  | Cost of vaccine, adults’ type | 91,541 | 126,627 | 35,085 |  |
|  | Proportion of seeking healthcare in urban area | 93,991 | 120,396 | 26,405 |  |
| Heilongjiang | Symptomatic influenza rate among children aged 5-14 y | 100,896 | 195,730 | 94,834 | 5238 |
|  | Influenza-associated excess respiratory mortality | 91,363 | 171,194 | 79,830 |  |
|  | Odds ratio of vaccination effectiveness | 119,570 | 196,951 | 77,382 |  |
|  | Symptomatic influenza rate among children aged < 5 y | 114,058 | 165,386 | 51,328 |  |
|  | Proportion of seeking healthcare in urban area | 127,841 | 170,005 | 42,165 |  |
| Shanghai | Influenza-associated excess respiratory mortality | -109,918 | -82,928 | 26,990 | 22,768 |
|  | QALY loss of self-medication | -107,210 | -87,463 | 19,746 |  |
|  | Duration of outpatient | -106,397 | -89,862 | 16,536 |  |
|  | Proportion of high-risk children aged 5-14 y | -104,101 | -87,706 | 16,395 |  |
|  | Symptomatic influenza rate among children aged < 5 y | -104,525 | -90,586 | 13,938 |  |
| Jiangsu | Influenza-associated excess respiratory mortality | -346 | 41,067 | 41,413 | 17,893 |
|  | Odds ratio of vaccination effectiveness | 19,157 | 60,092 | 40,935 |  |
|  | Symptomatic influenza rate among children aged 5-14 y | 15,231 | 49,968 | 34,737 |  |
|  | Symptomatic influenza rate among children aged < 5 y | 14,429 | 48,320 | 33,891 |  |
|  | Proportion of seeking healthcare in urban area | 22,168 | 48,391 | 26,223 |  |
| Zhejiang | Influenza-associated excess respiratory mortality | -25,442 | 5868 | 31,310 | 15,580 |
|  | Odds ratio of vaccination effectiveness | -18,571 | 3847 | 22,418 |  |
|  | Symptomatic influenza rate among children aged < 5 y | -21,052 | -4657 | 16,395 |  |
|  | Symptomatic influenza rate among children aged 5-14 y | -18,413 | -4738 | 13,676 |  |
|  | Proportion of seeking healthcare in urban area | -15,909 | -3982 | 11,927 |  |
| Anhui | Symptomatic influenza rate among children aged 5-14 y | 27,445 | 67,871 | 40,426 | 8468 |
|  | Influenza-associated excess respiratory mortality | 24,601 | 62,223 | 37,622 |  |
|  | Odds ratio of vaccination effectiveness | 35,411 | 71,534 | 36,123 |  |
|  | Cost of vaccine, adults’ type | 40,746 | 58,031 | 17,285 |  |
|  | Proportion of seeking healthcare in urban area | 40,869 | 56,296 | 15,426 |  |
| Fujian | Influenza-associated excess respiratory mortality | -62,026 | 151,152 | 213,178 | 15,509 |
|  | Odds ratio of vaccination effectiveness | 25,504 | 81,689 | 56,185 |  |
|  | Symptomatic influenza rate among children aged 5-14 y | 12,196 | 44,342 | 32,146 |  |
|  | Cost of vaccine, adults’ type | 35,045 | 58,729 | 23,684 |  |
|  | Proportion of seeking healthcare in urban area | 35,644 | 56,265 | 20,621 |  |
| Jiangxi | Influenza-associated excess respiratory mortality | 68,176 | 480,405 | 412,230 | 7,696 |
|  | Symptomatic influenza rate among children aged 5-14 y | 261,489 | 458,397 | 196,908 |  |
|  | Odds ratio of vaccination effectiveness | 297,049 | 477,987 | 180,938 |  |
|  | Cost of vaccine, adults’ type | 322,034 | 412,945 | 90,911 |  |
|  | QALY loss of self-medication | 318,406 | 396,193 | 77,787 |  |
| Shandong | Influenza-associated excess respiratory mortality | 13,903 | 122,029 | 108,126 | 10,228 |
|  | Odds ratio of vaccination effectiveness | 36,245 | 86,059 | 49,814 |  |
|  | Symptomatic influenza rate among children aged 5-14 y | 28,777 | 78,252 | 49,475 |  |
|  | Cost of vaccine, adults’ type | 44,685 | 65,734 | 21,050 |  |
|  | Proportion of seeking healthcare in urban area | 44,995 | 63,642 | 18,648 |  |
| Henan | Symptomatic influenza rate among children aged 5-14 y | 79,691 | 191,334 | 111,643 | 8163 |
|  | Odds ratio of vaccination effectiveness | 104,193 | 176,317 | 72,124 |  |
|  | Influenza-associated excess respiratory mortality | 80,578 | 150,462 | 69,885 |  |
|  | Cost of vaccine, adults’ type | 113,562 | 151,360 | 37,798 |  |
|  | Symptomatic influenza rate among children aged < 5 y | 107,307 | 143,785 | 36,477 |  |
| Hubei | Influenza-associated excess respiratory mortality | 23,254 | 73,815 | 50,561 | 11,203 |
|  | Odds ratio of vaccination effectiveness | 33,914 | 76,859 | 42,945 |  |
|  | Symptomatic influenza rate among children aged < 5 y | 27,713 | 64,105 | 36,392 |  |
|  | Cost of vaccine, adults’ type | 40,513 | 60,400 | 19,887 |  |
|  | Proportion of seeking healthcare in urban area | 39,993 | 59,548 | 19,555 |  |
| Hunan | Influenza-associated excess respiratory mortality | 67,994 | 388,572 | 320,578 | 8329 |
|  | Odds ratio of vaccination effectiveness | 200,999 | 335,702 | 134,703 |  |
|  | Symptomatic influenza rate among children aged 5-14 y | 198,297 | 279,514 | 81,217 |  |
|  | Cost of vaccine, adults’ type | 220,502 | 285,898 | 65,396 |  |
|  | Symptomatic influenza rate among children aged < 5 y | 205,056 | 262,433 | 57,376 |  |
| Guangdong | Influenza-associated excess respiratory mortality | -38,363 | 12,604 | 50,967 | 13,632 |
|  | Odds ratio of vaccination effectiveness | -21,179 | -376 | 20,804 |  |
|  | Symptomatic influenza rate among children aged 5-14 y | -19,660 | -8955 | 10,705 |  |
|  | Cost of vaccine, adults’ type | -17,518 | -9075 | 8443 |  |
|  | Proportion of high-risk children aged < 5 y | -21,172 | -12,869 | 8303 |  |
| Guangxi | Influenza-associated excess respiratory mortality | -14,598 | 138,574 | 153,173 | 6219 |
|  | Odds ratio of vaccination effectiveness | 13,987 | 50,741 | 36,754 |  |
|  | Cost of vaccine, adults’ type | 19,042 | 37,587 | 18,545 |  |
|  | Symptomatic influenza rate among children aged 5-14 y | 17,447 | 35,768 | 18,321 |  |
|  | Proportion of high-risk children aged < 5 y | 12,558 | 29,179 | 16,621 |  |
| Hainan | Influenza-associated excess respiratory mortality | -21,943 | 21,831 | 43,774 | 8180 |
|  | Odds ratio of vaccination effectiveness | -11,171 | 8550 | 19,721 |  |
|  | Symptomatic influenza rate among children aged 5-14 y | -9500 | 128 | 9628 |  |
|  | Cost of vaccine, adults’ type | -8261 | 1184 | 9445 |  |
|  | Proportion of high-risk children aged < 5 y | -11,931 | -3101 | 8830 |  |
| Chongqing | Influenza-associated excess respiratory mortality | -9986 | 141,838 | 151,824 | 10,977 |
|  | Odds ratio of vaccination effectiveness | 51,749 | 108,893 | 57,144 |  |
|  | Symptomatic influenza rate among children aged 5-14 y | 43,538 | 100,384 | 56,847 |  |
|  | Cost of vaccine, adults’ type | 60,454 | 87,109 | 26,655 |  |
|  | Proportion of seeking healthcare in urban area | 60,344 | 85,465 | 25,121 |  |
| Sichuan | Influenza-associated excess respiratory mortality | -3669 | 32,012 | 35,681 | 8074 |
|  | Odds ratio of vaccination effectiveness | 12,875 | 36,589 | 23,715 |  |
|  | Symptomatic influenza rate among children aged 5-14 y | 11,774 | 28,653 | 16,879 |  |
|  | Symptomatic influenza rate among children aged < 5 y | 11,524 | 27,636 | 16,112 |  |
|  | Cost of vaccine, adults’ type | 16,260 | 27,909 | 11,649 |  |
| Guizhou | Influenza-associated excess respiratory mortality | -18,615 | 69,487 | 88,102 | 6722 |
|  | Odds ratio of vaccination effectiveness | 1877 | 27,579 | 25,702 |  |
|  | Proportion of high-risk children aged < 5 y | -1516 | 13,067 | 14,582 |  |
|  | Cost of vaccine, adults’ type | 5467 | 18,297 | 12,830 |  |
|  | Symptomatic influenza rate among children aged < 5 y | 1907 | 11,027 | 9120 |  |
| Yunnan | Influenza-associated excess respiratory mortality | -16,220 | 177,968 | 194,188 | 6940 |
|  | Odds ratio of vaccination effectiveness | 9658 | 47,304 | 37,646 |  |
|  | Proportion of high-risk children aged < 5 y | 5493 | 25,973 | 20,480 |  |
|  | Cost of vaccine, adults’ type | 15,025 | 33,535 | 18,510 |  |
|  | Symptomatic influenza rate among children aged < 5 y | 11,954 | 26,090 | 14,136 |  |
| Tibet | Influenza-associated excess respiratory mortality | -19,392 | 43,197 | 62,588 | 7079 |
|  | Odds ratio of vaccination effectiveness | -7814 | 18,910 | 26,724 |  |
|  | Proportion of high-risk children aged < 5 y | -12,221 | 4157 | 16,378 |  |
|  | Symptomatic influenza rate among children aged < 5 y | -9712 | 5002 | 14,714 |  |
|  | Cost of vaccine, adults’ type | -4194 | 9435 | 13,629 |  |
| Shaanxi | Influenza-associated excess respiratory mortality | 4471 | 41,023 | 36,551 | 9648 |
|  | Symptomatic influenza rate among children aged 5-14 y | 4721 | 33,865 | 29,143 |  |
|  | Odds ratio of vaccination effectiveness | 9261 | 35,682 | 26,421 |  |
|  | Cost of vaccine, adults’ type | 13,302 | 25,590 | 12,288 |  |
|  | Proportion of seeking healthcare in urban area | 13,370 | 24,577 | 11,207 |  |
| Gansu | Influenza-associated excess respiratory mortality | 708 | 50,844 | 50,137 | 4776 |
|  | Odds ratio of vaccination effectiveness | 17,632 | 42,023 | 24,391 |  |
|  | Symptomatic influenza rate among children aged 5-14 y | 18,262 | 31,108 | 12,846 |  |
|  | Cost of vaccine, adults’ type | 21,023 | 33,239 | 12,217 |  |
|  | Symptomatic influenza rate among children aged < 5 y | 16,712 | 27,104 | 10,392 |  |
| Qinghai | Influenza-associated excess respiratory mortality | -21,562 | 30,070 | 51,633 | 7090 |
|  | Odds ratio of vaccination effectiveness | -10,699 | 9479 | 20,178 |  |
|  | Symptomatic influenza rate among children aged 5-14 y | -10,062 | 2858 | 12,920 |  |
|  | Cost of vaccine, adults’ type | -7895 | 2216 | 10,111 |  |
|  | Proportion of high-risk children aged < 5 y | -10,368 | -2670 | 7698 |  |
| Ningxia | Influenza-associated excess respiratory mortality | -25,231 | 28,460 | 53,692 | 7848 |
|  | Odds ratio of vaccination effectiveness | -13,716 | 6858 | 20,574 |  |
|  | Symptomatic influenza rate among children aged 5-14 y | -12,165 | -1400 | 10,764 |  |
|  | Cost of vaccine, adults’ type | -10,610 | -938 | 9672 |  |
|  | Proportion of high-risk children aged < 5 y | -14,711 | -5230 | 9480 |  |
| Xinjiang | Influenza-associated excess respiratory mortality | -22,193 | 77,824 | 100,017 | 7858 |
|  | Odds ratio of vaccination effectiveness | -5316 | 25,853 | 31,169 |  |
|  | Symptomatic influenza rate among children aged 5-14 y | -6651 | 17,901 | 24,553 |  |
|  | Symptomatic influenza rate among children aged < 5 y | -5474 | 13,234 | 18,708 |  |
|  | Cost of vaccine, adults’ type | -1802 | 15,914 | 17,716 |  |


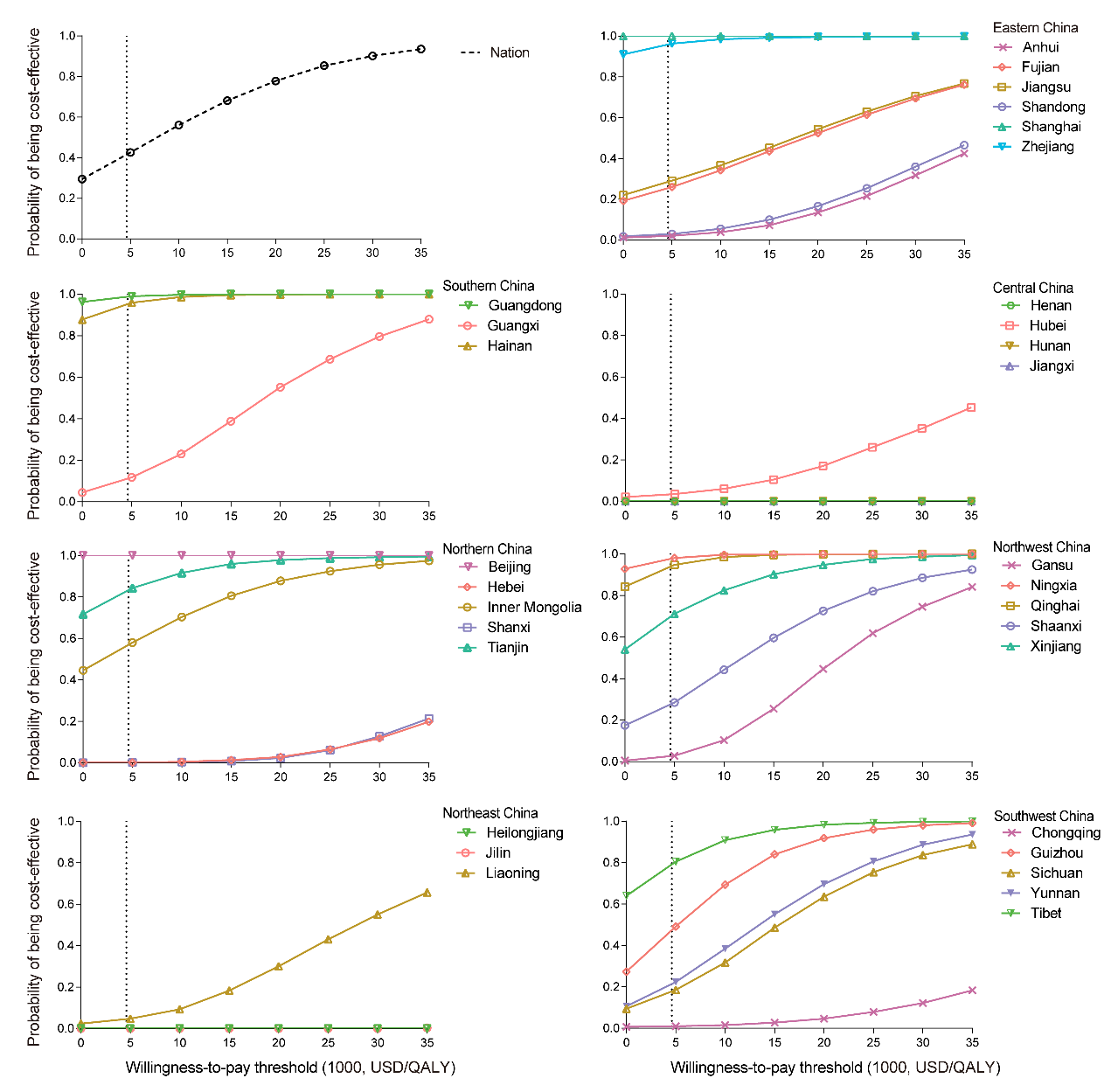


**Figure S3. Cost-effectiveness acceptability curves at national and provincial level (Woods et al. threshold)**


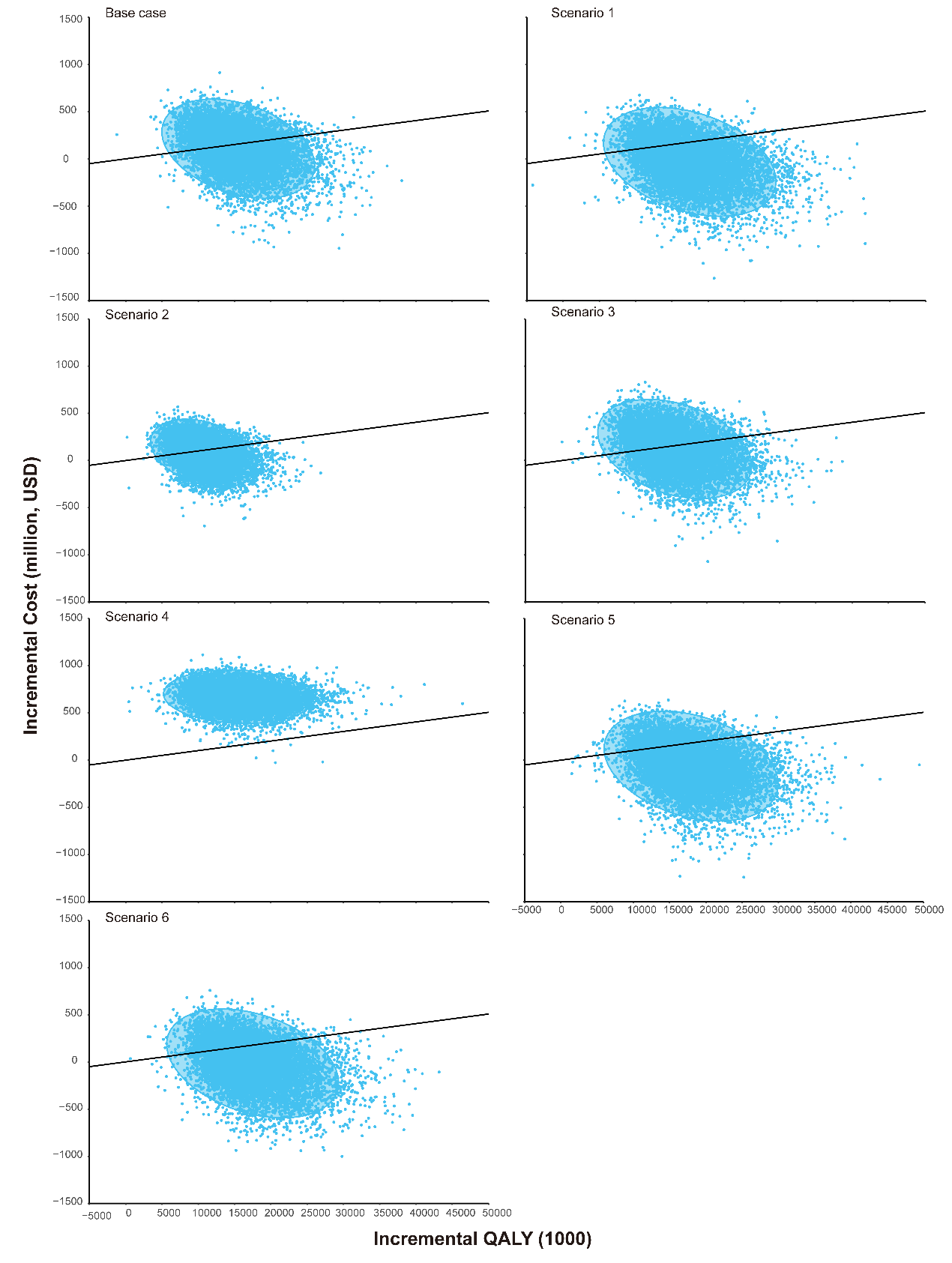


**Figure S4. Monte Carlo simulation results in various scenarios^#^**

^#^ the black line was willingness-to-pay threshold (GDP per capita).


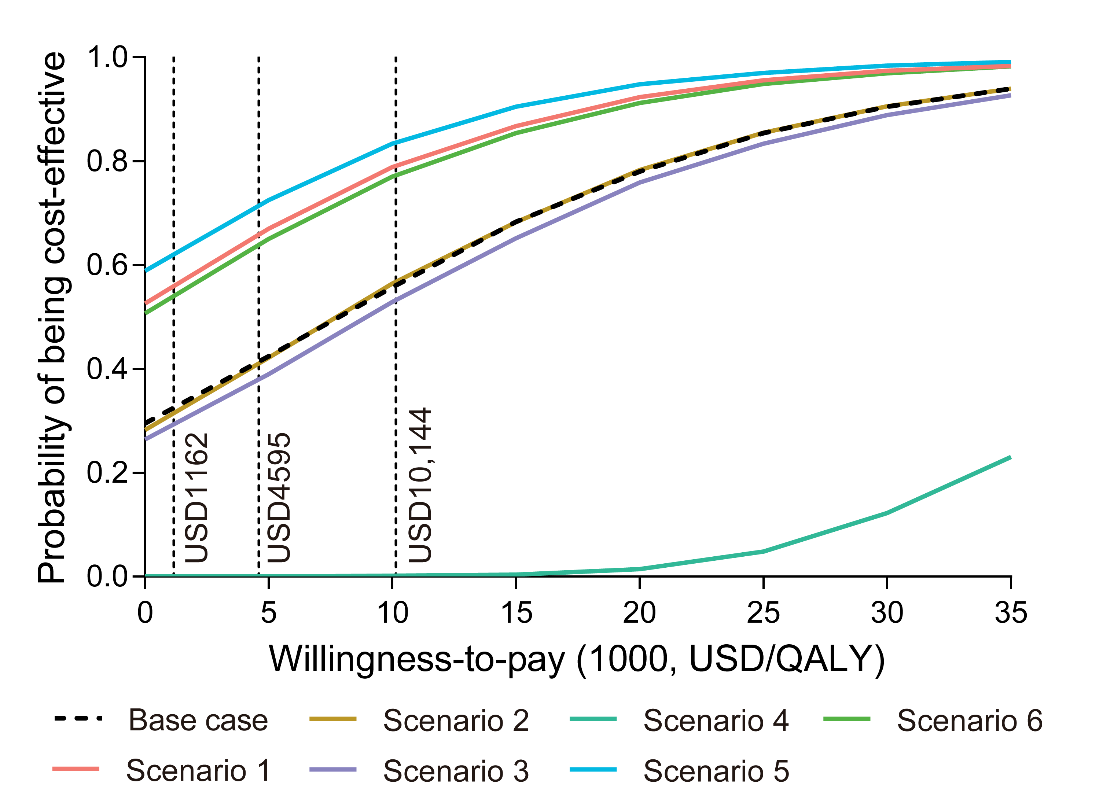


**Figure S5.** **Cost-effectiveness acceptability curves in various scenarios^#^**

^#^ Scenario 1: indirect effect; Scenario 2: low vaccination rate under fully-funded policy; Scenario 3: high proportions of side effects due to vaccinations; Scenario 4: healthcare perspective; Scenario 5: matched vaccine; Scenario 6: mixed situation.
